# Mitochondrion-Dependent Apoptosis Is Essential for Rickettsia parkeri Infection and Replication in Vector Cells

**DOI:** 10.1128/mSystems.01209-20

**Published:** 2021-03-16

**Authors:** Xin-Ru Wang, Nicole Y. Burkhardt, Timothy J. Kurtti, Jonathan D. Oliver, Lisa D. Price, Benjamin Cull, Cody J. Thorpe, Michalina Silva Thiel, Ulrike G. Munderloh

**Affiliations:** a Department of Entomology, University of Minnesota, St. Paul, Minnesota, USA; b School of Public Health, Division of Environmental Health Sciences, University of Minnesota, Minneapolis, Minnesota, USA; University of California, San Diego

**Keywords:** tick, apoptosis, mitochondria, *Rickettsia* pathogenesis, *Rickettsia*, tick-borne pathogens

## Abstract

Apoptosis is an innate immune response induced by infection in eukaryotes that contributes significantly to protection from pathogens. However, little is known about the role of apoptosis in the interactions of arthropod vectors with the rickettsiae that they transmit. *Rickettsia* spp. are vector-borne obligately intracellular bacteria and display different degrees of virulence in their eukaryotic hosts. In this study, we found that infection with Rickettsia parkeri (*Rp*) activated the apoptosis pathway in an *Amblyomma americanum* tick cell line (AAE2), as evidenced by the loss of phospholipid membrane asymmetry and DNA fragmentations. Additionally, infection with *Rp* also led to apoptosis activation in cell lines of different tick species. Interestingly, suppressing apoptosis decreased *Rp* infection and replication, while the activation of apoptosis increased *Rp* accumulation at the early stage of infection. Moreover, mitochondrion-dependent apoptosis was essential for *Rp* infection and replication in vector cells, and apoptosis induction required intracellular rickettsia replication. We further showed that *Rp* utilizes two different survival strategies to modulate apoptosis in the arthropod vectors and mammalian host cells. There was no direct correlation between apoptosis activation in vector cells and rickettsial pathogenicity. These novel findings indicate a possible mechanism whereby apoptosis facilitates infection and replication of a *Rickettsia* sp. in an arthropod vector. These results contribute to our understanding of how the vector's responses to pathogen infection affect pathogen replication and therefore transmission.

**IMPORTANCE** Rickettsioses, infections caused by the genus *Rickettsia*, are among the oldest known infectious diseases. Ticks are essential arthropod vectors for rickettsiae, and knowledge about the interactions between ticks, their hosts, and pathogens is fundamental for identifying drivers of tick-borne rickettsioses. Despite the rapid development in apoptosis research with rickettsiae, little is known regarding the role of apoptosis in the interactions between *Rickettsia* spp., vertebrate hosts, and arthropod vectors. Here, we demonstrated that mitochondrion-dependent apoptosis is essential for rickettsial infection and replication in vector cells and that apoptosis induction requires intracellular rickettsial replication. However, rickettsial pathogenicity is not linked with apoptosis activation in tick cells. Our findings improve understanding of the apoptosis mechanism in arthropods exploited by rickettsiae and also the potential to discover specific targets for new vaccines and drugs to prevent or treat rickettsial infections.

## INTRODUCTION

Rickettsioses, infections caused by obligate intracellular bacteria belonging to the genus *Rickettsia* (family *Rickettsiaceae*, order *Rickettsiales*), are among the oldest known infectious diseases (1). Currently, more than 30 identified species in the genus *Rickettsia* and various uncharacterized strains cause diseases and exert an enormous impact on human, animal, and ecosystem health globally (2). Members of the genus *Rickettsia* are classified into four or more groups, including the spotted fever group (SFG), the typhus group (TG), the transitional group (TRG), and the ancestral group (AG), according to phylogenetic characteristics (3, 4). One of the SFG members is Rickettsia rickettsii, the causal agent of Rocky Mountain spotted fever, which is the most severe of all tick-borne rickettsioses and remains the most frequently reported fatal tick-borne infection in the United States (5). Previous studies indicated that R. rickettsii affects diverse cellular functions, such as directly influencing gene transcription of the NF-*κ*B pathway, manipulating the host cell cytoskeleton, activating signaling pathways, and regulating programmed cell death for subsequent intracellular multiplication (6–9). Members of the SFG rickettsiae have different effects on eukaryotic cells, and unlike for R. rickettsii, little is known regarding how they survive in the arthropod vectors and facilitate their own multiplication and transmission.

Apoptosis is a highly regulated process that maintains homeostatic cellular balance and is also well studied as a defense mechanism in immune reactions (10). The evolutionarily conserved apoptosis features include blebbing, cellular shrinkage, nuclear fragmentation, chromosomal DNA fragmentation, global mRNA decay, and activation of caspases (11). A growing number of discoveries reveal that pathogens have evolved a series of molecular mechanisms to modulate apoptosis and overcome a final challenge: how to stay alive, replicate, and spread (12). In some cases, induction of apoptosis promotes bacterial survival by increasing tumor necrosis factor alpha (TNF-α)-producing cells, activating caspase-1, binding caspase, and activating protease, which inhibits cellular survival pathways and directly releases cytochrome *c*, thus downregulating antiapoptotic protein expression (13–15). In other cases, prevention of apoptosis favors bacterial infection and replication by protecting mitochondrial potential and inhibiting cytochrome *c* release. This activates survival pathways and directly regulates cytokine synthesis, thus blocking caspase-activation and upregulating antiapoptotic molecular signals (16–19). The dual activity of bacteria in manipulating apoptosis depends on the bacterial taxon, the duration of infection, the host cell type, and other factors.

Among pathogenic prokaryotes, the facultatively and obligately intracellular bacteria face a particularly difficult problem: overcoming host cell barriers to invasion, replication, and transmission (20). To meet those challenges, some have developed a variety of strategies modulating host defenses, including apoptosis, such as Shigella flexneri, Listeria monocytogenes, Legionella pneumophila, Anaplasma phagocytophilum, and Mycobacterium tuberculosis (21). A prime example of intracellular bacteria regulating apoptosis is A. phagocytophilum (*Rickettsiales*: *Anaplasmataceae*). It does so by preventing cytochrome *c* release, blocking cell surface Fas, and upregulating the Bcl-2 family members (22, 23). Similarly, R. rickettsii protects infected vascular endothelial cells from apoptosis through the activation of the NF-κB signaling pathway, targeting anti-/proapoptotic proteins, and using the cIAP2-independent mechanism (24–26). However, the role of apoptosis in the interactions between *Rickettsia* spp., vertebrate host cells, and arthropod host cells is poorly understood and not as well characterized as for A. phagocytophilum.

Since the discovery of salivary gland apoptosis in ticks (27, 28), a growing body of evidence supports the significance of apoptosis in maintenance of multicellular organisms and host-pathogen interactions (29–31). Nevertheless, little is known regarding apoptosis mechanisms in arthropod vectors and how they affect pathogen transmission. For example, questions regarding whether other *Rickettsia* spp. trigger or inhibit apoptosis in their arthropod vectors, and what the apoptotic molecular responses are, remain unanswered. We do not know the key *Rickettsia* effectors involved in modulating the apoptosis responses in various hosts and vectors. To address these questions, we utilized one SFG species, Rickettsia parkeri (*Rp*), that causes mild or moderate disease in North and South America (32) and whose life cycle and ability to invade host cells are similar to those of R. rickettsii (33, 34). Here, we show that mitochondrion-dependent apoptosis is essential for *Rp* infection and replication in vector cells and that apoptosis induction requires intracellular rickettsial replication. However, rickettsial pathogenicity is not linked to apoptosis activation in tick cells. These results improve understanding of the apoptosis mechanism in arthropod vectors exploited by rickettsiae and fill a critical void in our knowledge of vector-rickettsia-host molecular interactions.

## RESULTS

### Apoptosis is activated in tick cells during *Rp* infection.

Loss of plasma membrane asymmetry is one of the earliest morphological features of apoptosis (35). Annexin V, a phospholipid-binding protein retaining its high affinity for phospholipid phosphatidylserine (PS), is broadly used in conjunction with propidium iodide (PI) for analyzing cells undergoing apoptosis (36). We determined whether *Rp* infection can trigger apoptosis in a cell line from a suspected natural vector, the lone star tick, Amblyomma americanum. This tick has been shown to naturally harbor *Rp* and to acquire it during cofeeding with closely related Gulf Coast ticks, Amblyomma maculatum, in a laboratory setting (37). AAE2 cells infected with wild-type *Rp* were cultured for 4 days, and uninfected cells served as controls. Infected AAE2 cells were then double-stained with annexin V-fluorescein isothiocyanate (FITC) (green signal) and PI (red signal) to distinguish cells in different apoptosis phases by confocal microscopy.

**FIG 1:**
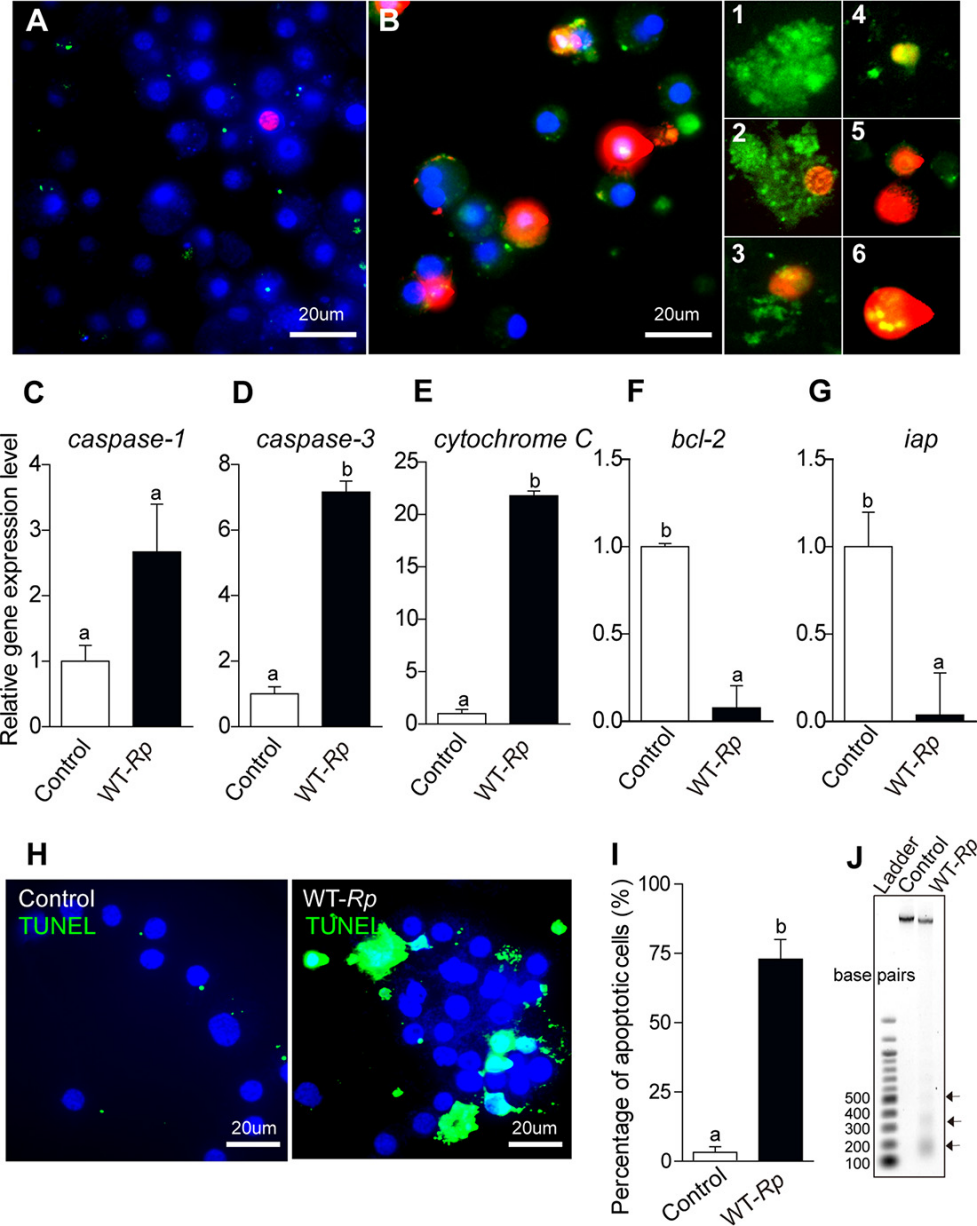
Rickettsia parkeri (*Rp*) infection induces apoptosis in AAE2 cells. Cells infected with *Rp* after 4 days were fixed and stained by a combination of fluorescein annexin V-FITC (green) and propidium iodide (PI, red). Blue DAPI staining corresponds to the nuclei. (A) Uninfected AAE2 cells. (B) Different labeling patterns of *Rp*-infected cells with both annexin V-FITC and PI-positive cells. 1, early apoptotic cells, annexin V-FITC positive and PI negative; 2 to 4, necrotic or late apoptotic cells, both annexin V-FITC and PI positive; 5 and 6, dead cells, annexin V-FITC negative and PI positive. (C to G) The relative expression level of apoptosis-related genes was tested by qRT-PCR in uninfected (control) and wild-type-*Rp*-infected AAE2 cells; expression of *gapdh* was used as the internal control. (H) *Rp*-infected AAE2 cells were fixed and labeled with TUNEL (green). Bar, 20 μm. Blue DAPI staining corresponds to the nuclei. (I) Percent apoptotic cells (number of TUNEL-positive cells/number of DAPI-positive cells) in different fields. (J) DNA laddering assay. *Rp* induced apoptotic DNA fragmentation in AAE2 cells by gel electrophoresis. A 100-bp ladder was the marker used to indicate the internucleosomal DNA fragments (arrows). In panels C, D, E, F, G, and I, data are means and standard deviations (SD), and different letters above the columns indicate significant differences.

More apoptotic cells were observed after *Rp* infection than in uninfected AAE2 cells (Fig. 1A and B; panel 1, early phase of apoptosis, annexin V-FITC positive and PI negative; panels 2 to 4, late phase of apoptosis, both annexin V-FITC and PI positive; panels 5 and 6, terminal [dead cells] phase of apoptosis, annexin V-FITC negative and PI positive). Furthermore, we examined the expression level of 5 apoptosis-related genes in AAE2 cells (*caspase-1*, *caspase-3*, *cytochrome c*, *b-cell lymphoma-2*, and *inhibitors of apoptosis protein*) after *Rp* infection (Fig. 1C to G). Caspase-1 and caspase-3 act as initiator and effector caspases to activate apoptosis (38); cytochrome *c* is involved in the initiation of apoptosis by its release from mitochondria into the cytosol (39); bcl-2 protein inhibits apoptosis via interactions with proapoptotic proteins; and inhibitors of apoptosis (Iap) can act to block caspases (40). Real-time PCR results showed that the expression level of caspase-3 and cytochrome *c* genes increased, while *bcl-2* and *iap* expression levels were significantly reduced upon *Rp* infection relative to control. To further verify that apoptosis was induced by *Rp* infection, DNA fragmentation in the AAE2 cells was examined using terminal deoxynucleotidyl transferase (TdT)-mediated dUTP nick-end labeling (TUNEL) (41). This method also showed that more apoptotic cells were present in *Rp*-infected cultures than in controls (Fig. 1H and I), and gel electrophoresis demonstrating DNA laddering indicated the presence of internucleosomal DNA fragments in *Rp*-infected AAE2 cells as well (Fig. 1J). Thus, these data strongly suggest that apoptosis is activated during the later stage of *Rp* infection (4 days postinfection [p.i.]) in AAE2 cells.

Cell-type-specific differences in the host-pathogen interaction led us to suspect that apoptosis induction might be specific to its vector tick cell line. To test this hypothesis, we used cell lines from three other tick species not considered vectors of *Rp*, ISE6 (Ixodes scapularis; black-legged ticks), BME26 (Boophilus microplus; cattle tick), and IRE11 (Ixodes ricinus; European sheep tick), to monitor apoptosis during the later stage of *Rp* infection (4 days p.i.). Unexpectedly, we found that results were consistent with those obtained with AAE2 cells: infection with *Rp* led to apoptosis activation, as indicated by TUNEL and DNA laddering gel assay (see Fig. S1 in the supplemental material). Colocalization experiments with TUNEL (green signals) and pRAM18dSFA-transformed *Rp* (red signals) on the same sample showed that apoptosis was activated in the presence of *Rp* (Fig. S2). These results confirmed that the activation of apoptosis is strongly correlated with *Rp* infection in cell lines from different tick species, suggesting a fundamentally conserved response.

10.1128/mSystems.01209-20.3 FIG S1 *Rp* infection induces apoptosis in cells from different species of ticks. (A to C) Ixodes scapularis ISE6 cells. (D to F) Ixodes ricinus IRE11 cells. (G to I) *Rhipicephalus* (*Boophilus*) *microplus* BME26 cells. Wild-type *Rp*-infected infected ISE6 (A), IRE11 (D), and BME26 (G) cells after four days were fixed and labeled with TUNEL (green). Bar, 50 μm. Blue DAPI staining corresponds to the nuclei. Percent apoptotic ISE6 (B), IRE11 (E), and BME26 (H) cells (number of TUNEL-positive cells/number of DAPI-positive cells) in different fields. DNA laddering assay. *Rp*-induced apoptotic DNA fragmentation in ISE6 (C), IRE11 (F), and BME26 (I) cells was assessed by gel electrophoresis. Image of gel after electrophoretic separation of internucleosomal DNA fragments, indicated by arrows. The ladder was a 1-kb plus DNA ladder size marker. In panels B, E, and H, data are means and SD, and different letters above the columns indicate significant differences. Download FIG S1, JPG file, 0.1 MB. Copyright © 2021 Wang et al. 2021 Wang et al. https://creativecommons.org/licenses/by/4.0/ This content is distributed under the terms of the Creative Commons Attribution 4.0 International license.

10.1128/mSystems.01209-20.4 FIG S2 Apoptosis is activated in the presence of *Rp*. pRAM18dSFA-transformed *Rp* (red)-infected ISE6, IRE11, AAE2, and BME26 cells were fixed and labeled with TUNEL (green). Bar, 20 μm. Blue DAPI staining corresponds to the nuclei. Download FIG S2, TIF file, 1.5 MB. Copyright © 2021 Wang et al. 2021 Wang et al. https://creativecommons.org/licenses/by/4.0/ This content is distributed under the terms of the Creative Commons Attribution 4.0 International license.

### Apoptosis is required for *Rp* infection and replication in tick cells.

**FIG 2:**
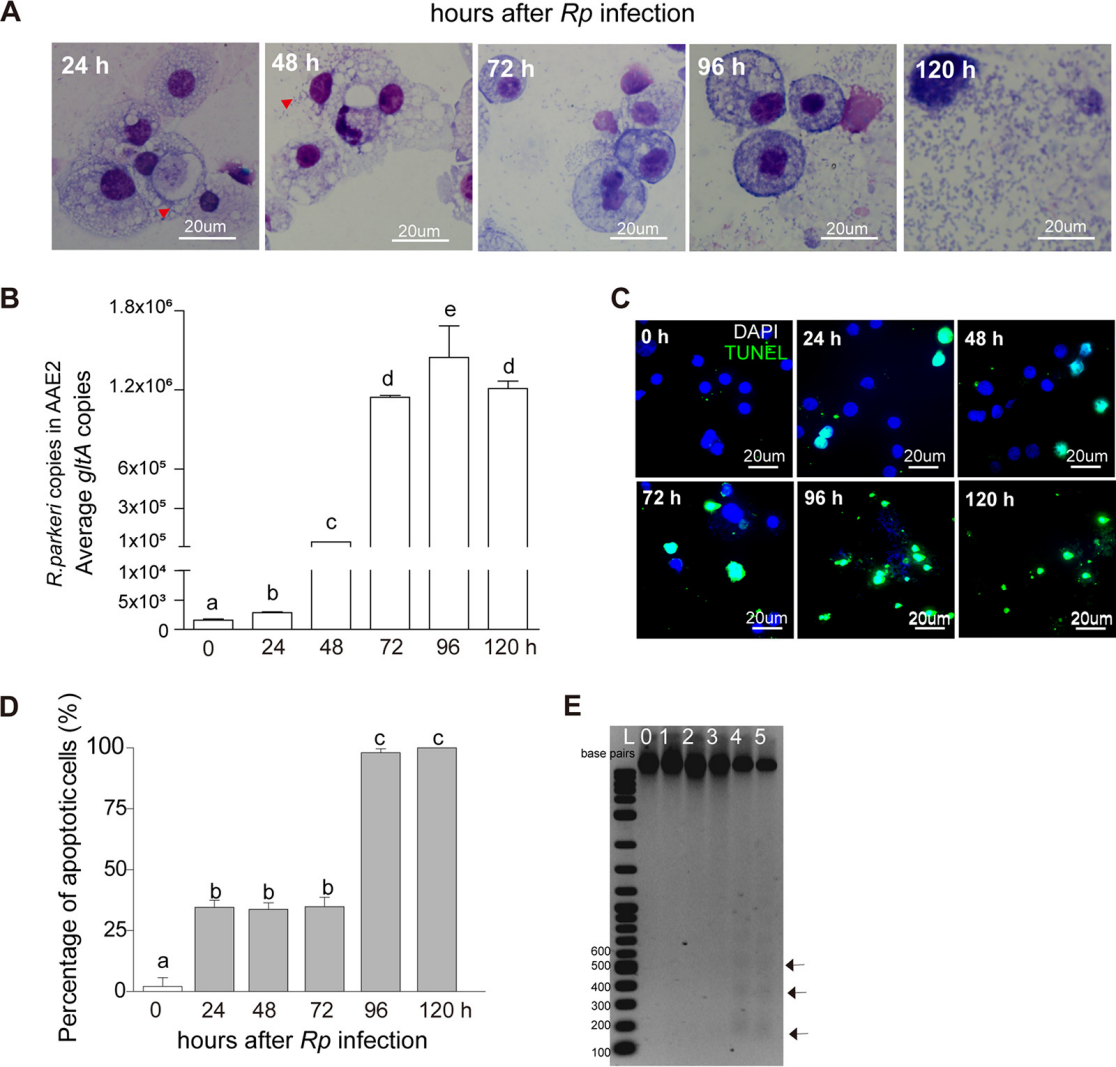
Time course of apoptosis activation in response to *Rp* infection. (A) Dynamics of *Rp* replication (indicated by red arrowheads) in AAE2 cells. Bar = 20 μm. (B) *Rp* genomic DNA abundance in AAE2 cells measured by qPCR using *gltA* as a reference. (C) AAE2 cells were fixed and labeled with TUNEL (green). Bar, 20 μm. Blue DAPI staining corresponds to the nuclei. (D) Percent apoptotic cells (number of TUNEL-positive cells/number of DAPI-positive cells) in different fields. Data are means and SD, and different letters above the columns indicate significant differences. (E) DNA laddering assay to assess *Rp*-induced apoptotic DNA fragmentation in AAE2 cells by gel electrophoresis. Internucleosomal DNA fragments are indicated by arrows. Lane L, 1-kb Plus DNA ladder.

Pathogen survival requires modulation of the host's immune response to allow colonization and replication and achieve transmission to a new host (42). Apoptosis is one of the programmed cell death pathways affected by intracellular bacterial pathogens such as R. rickettsii and is thought to mediate successful colonization of vertebrate host cells (24). To determine the possible effect of apoptosis on *Rp* infection and replication, we infected AAE2 cells with host cell-free wild-type *Rp* (multiplicity of infection [MOI] = 1) and collected samples at different time points. Giemsa staining (Fig. 2A) illustrated the replication dynamics of *Rp* for 120 h postinfection. Additionally, we determined *Rp* genomic DNA abundance using quantitative PCR (qPCR), which confirmed results using Giemsa-stained cell preparations; i.e., the level of *Rp* genomic DNA increased rapidly and peaked at 120 h (Fig. 2B). These results supported our observation that *Rp* replicated and accumulated within AAE2 cells.

The observation that *Chlamydia* exerts both proapoptotic and antiapoptotic effects at different infection stages suggested a specific strategy of intracellular bacteria in manipulating host cell apoptosis (43, 44). To determine whether pro- and antiapoptotic activities are manifested during different stages of *Rp* infection, we tested for the presence of apoptotic cells from 0 h to 120 h p.i. Compared to the later stage of infection (96 and 120 h), the percentage of apoptotic tick cells was significantly lower in the early infection stage (from 24 to 72 h) (Fig. 2C and D). In addition, the DNA laddering assay detected internucleosomal DNA fragments at 96 h and 120 h (Fig. 2E), corroborating activation of the apoptosis pathway at the later stage. Interestingly, *Rp* genomic DNA content began to increase after 24 h p.i., corresponding to the time when apoptosis was activated. We also monitored apoptosis responses after *Rp* time course infection in cell lines from three other tick species (Fig. S3). Similar to previous observations for AAE2 cells, more apoptotic cells were observed in BME26 and ISE6 at the later stage of infection (96 and 120 h) relative to the early stage of infection (from 24 to 72 h), whereas in IRE11, there was only a slight increase of the percentage of apoptotic cells after 72 h p.i.; however, the difference between the early and later stage of infection was quite consistent, as seen in AAE2 cells. These results suggested that *Rp* might display antiapoptotic activity during the early stage of the infection but exerts a proapoptotic activity in the later stage of infection.

10.1128/mSystems.01209-20.5 FIG S3 Time course of apoptosis activation in response to *Rp* infection. (A, C, and E) IRE11 (A), ISE6 (C), and BME26 (E) cells were infected with host cell-free *Rp* (MOI = 1), fixed, and labeled with TUNEL (green). Bar, 20 μm. Blue DAPI staining corresponds to the nuclei. (B, D, and F) Percent apoptotic cells (number of TUNEL-positive cells/number of DAPI-positive cells) in different fields. Download FIG S3, JPG file, 0.3 MB. Copyright © 2021 Wang et al. 2021 Wang et al. https://creativecommons.org/licenses/by/4.0/ This content is distributed under the terms of the Creative Commons Attribution 4.0 International license.

**FIG 3:**
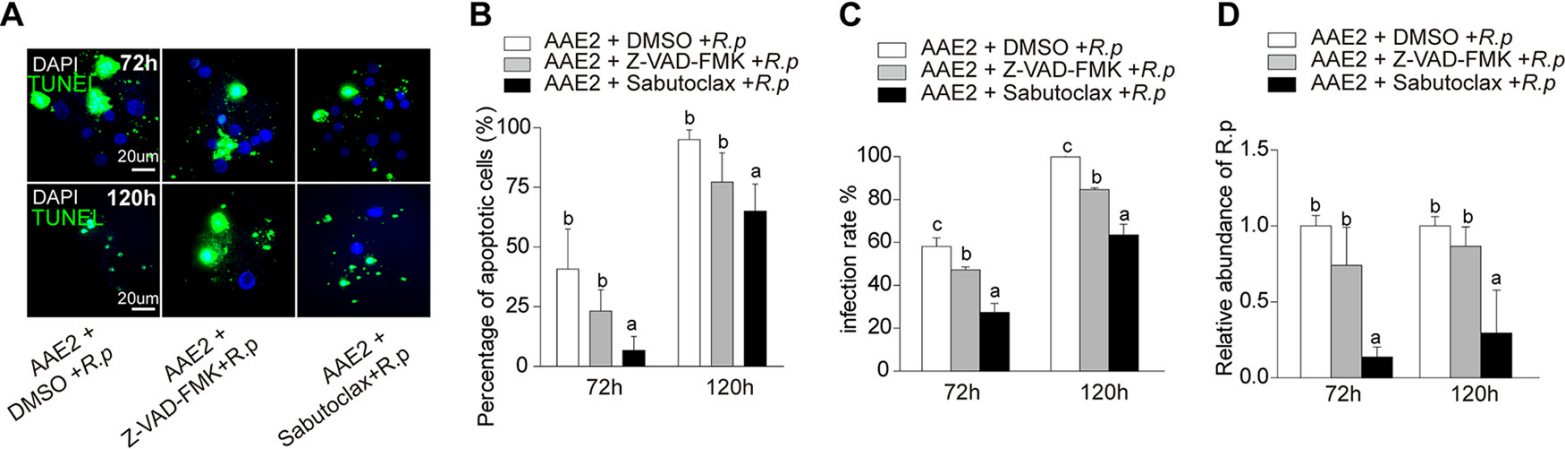
Effects of apoptosis inhibitors on *Rp* infection and replication. AAE2 cells were treated with the apoptosis inhibitor Z-VAD-FMK or sabutoclax or with a DMSO control and then infected with host cell-free *Rp* for 72 h and 120 h before being collected for analysis. (A) *Rp*-infected AAE2 cells were fixed and labeled with TUNEL (green) and DAPI (blue). Bar, 20 μm. Blue DAPI staining corresponds to the nuclei. (B) Percent apoptotic cells (number of TUNEL-positive cells/number of DAPI-positive cells) in different fields of view. (C) *Rp* infection rate (number of *Rp*-infected cells/number of total cells) was determined by Giemsa staining. (D) The relative level of *Rp* genomic DNA abundance measured by qPCR using *gltA* as a reference control. In panels B, C, and D, data are means and SD, and different letters above the columns indicate significant differences (ANOVA, followed by Bonferroni test).

To determine whether apoptosis triggered by *Rp* would facilitate infection and replication of rickettsiae in tick cells, we tested for different infection rates and quantities of *Rp* by treating infected AAE2 cells with Z-VAD-FMK [carbobenzoxy-valyl-alanyl-aspartyl-(*O*-methyl)-fluoromethylketone; a pan-caspase inhibitor], sabutoclax (a pan-Bcl-2 inhibitor), or DMSO (dimethyl sulfoxide; a solvent control) (Fig. 3). The percentage of apoptotic tick cells upon sabutoclax treatment (Fig. 3A and B) was significantly lower than that in untreated AAE2 cells. At the same time, the infection rate (Fig. 3C) and the quantities of *Rp* (Fig. 3D) were sharply reduced upon sabutoclax treatment at 72 h and 120 h. In contrast, the number of apoptotic cells only slightly decreased in the presence of Z-VAD-FMK relative to untreated AAE2 (Fig. 3A and B). Interestingly, while Z-VAD-FMK treatment markedly reduced the infection rate, the numbers of *Rp* organisms were minimally reduced (Fig. 3C and D). Overall, these data were consistent with the TUNEL assay and suggested that inhibition of apoptosis led to a decrease of *Rp* replication in AAE2 cells. The different effects on *Rp* replication seen when two chemical inhibitors of apoptosis were applied, one that inhibits pan-caspases (Z-VAD-FMK) and another that interferes with Bcl-2 (sabutoclax), suggested that apoptosis mechanisms linked to Bcl-2 family proteins might regulate *Rp* replication.

**FIG 4:**
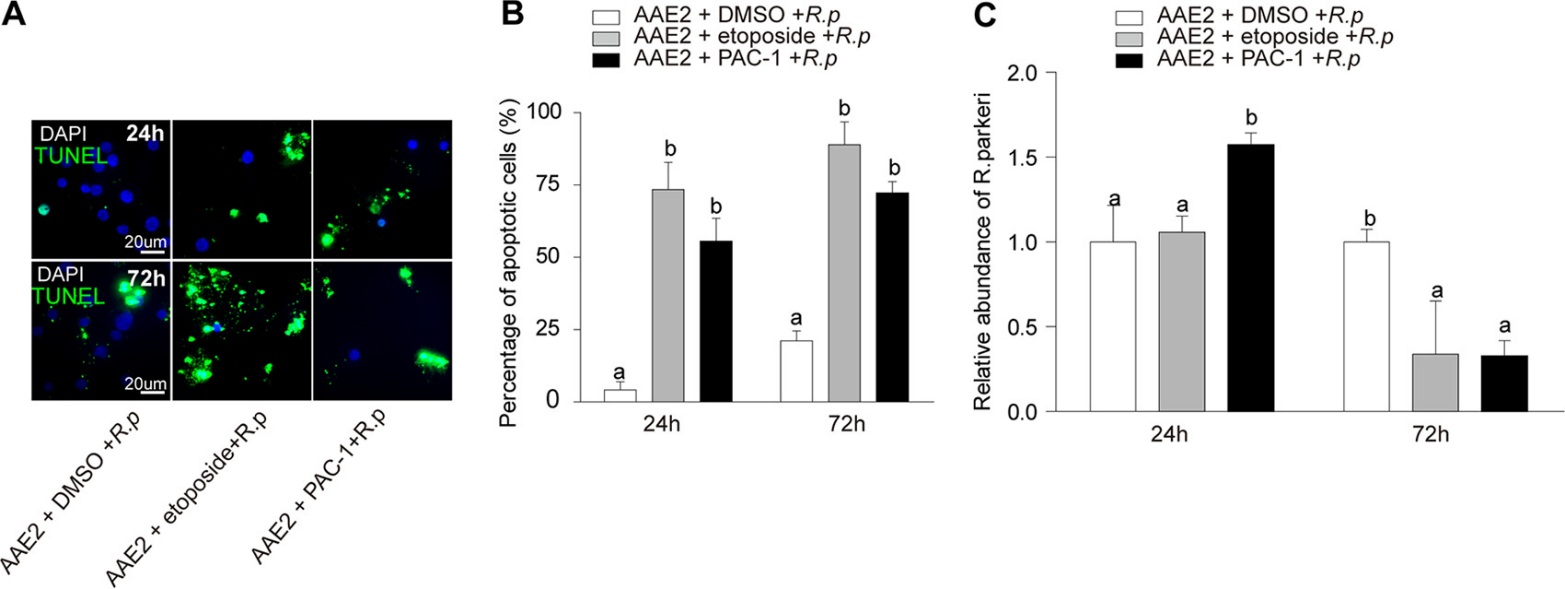
Effects of apoptosis inducers on *Rp* replication. AAE2 cells were treated with the apoptosis inducer PAC-1 or etoposide or with a DMSO control and then infected with host cell-free *Rp* for 72 h and 120 h before being collected for analysis. (A) *Rp*-infected AAE2 cells were fixed and labeled with TUNEL (green). Bar, 20 μm. Blue DAPI staining corresponds to the nuclei. (B) Percent apoptotic cells (number of TUNEL-positive cells/number of DAPI-positive cells) in different fields. (C) The relative level of *Rp* genomic DNA measured by qPCR and *gltA* as a reference control. In panels B and C, data are means and SD, and different letters above the columns indicate significant differences (ANOVA, followed by Bonferroni test).

To further verify the relationship between apoptosis and *Rp* replication, two chemical inducers, etoposide (an inhibitor of DNA topoisomerase II) (45, 46) and PAC-1 (a potent procaspase-3 activator) (47), were used to treat infected AAE2 cells (Fig. 4). The percentage of apoptotic cells (Fig. 4A and B) was significantly increased relative to untreated AAE2 after etoposide and PAC-1 treatment at 24 h and 72 h. Quantities of *Rp* were significantly increased only upon treatment with PAC-1 relative to untreated AAE2 cells at 24 h. In contrast, the quantities of *Rp* were unaffected by treatment with etoposide at 24 h (Fig. 4C), suggesting that caspase-3 might be the key player participating in *Rp*-induced apoptosis in tick cells. Interestingly, neither etoposide nor PAC-1 treatment affected the replication of *Rp* at 72 h, and both even reduced the quantities of *Rp*, perhaps reflecting *Rp* premature death due to excessive apoptosis of tick cells induced by chemical treatment. Thus, these data strongly indicate that early induction of apoptosis could facilitate *Rp* replication, while inhibition of apoptosis led to the decrease of *Rp* replication.

### The intrinsic apoptosis pathway is activated in response to *Rp* infection.

**FIG 5:**
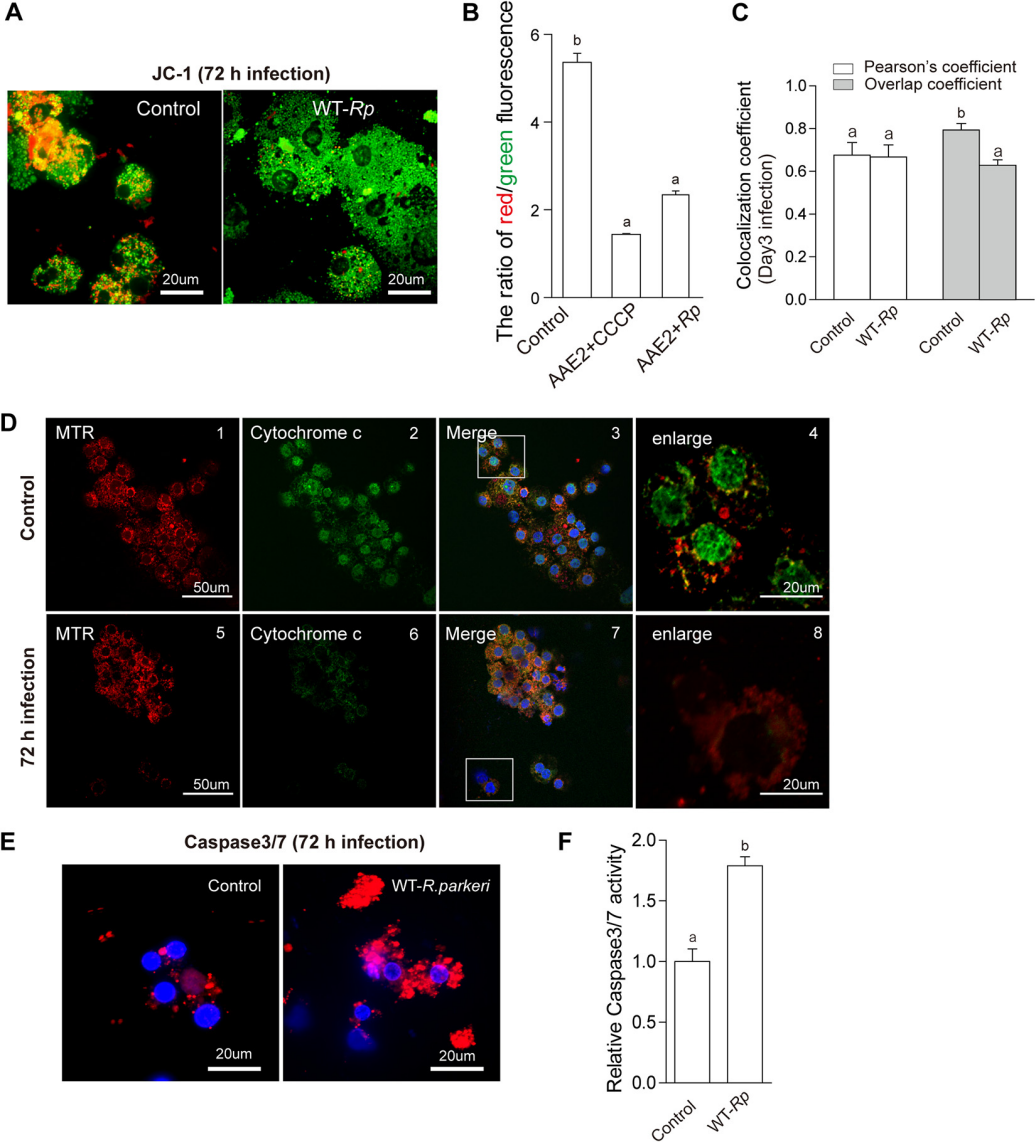
Mitochondrion-dependent apoptosis is required for *Rp* replication. Live cells were stained with MitoPT JC-1 (A and B). (A) Uninfected (left) and *Rp*-infected (right) AAE2 cells. (B) The ratio of red/green fluorescence in uninfected AAE2 cells (control), uninfected AAE2 cells treated with CCCP (positive control), and *Rp*-infected cells was measured using a fluorescence plate reader. Cytochrome *c* release from mitochondria into the cytosol after *Rp* infection in AAE2 (C and D). (C) Changes of Pearson’s correlation coefficient (PCC) and overlap coefficient according to Manders (MOC) resulted in a decrease in colocalization of cytochrome *c* and mitochondria. (D) Mitochondria (red signal) colocalized with FITC-labeled cytochrome *c* (green signal) in uninfected AAE2 cells (top row) and *Rp*-infected AAE2 cells (bottom row). The white boxes indicate the areas of the enlarged images. (E) *Rp*-infected AAE2 cells showed increasing red intensity as caspase activity and apoptosis progressed, relative to uninfected AAE2 cells. Bar, 20 μm. Blue DAPI staining corresponds to the nuclei. (F) Upregulated caspase-3/7 enzyme activity in *Rp*-infected AAE2 cells detected by the fluorescence plate reader is shown relative to uninfected AAE2 cells. In panels B (ANOVA, followed by Bonferroni test), C, and F, data are means and SD, and different letters above the columns indicate significant differences.

Changes in the mitochondrial membrane potential (ΔΨm) leading to the opening of the mitochondrial permeability transition pore are distinctive features of the intrinsic apoptosis pathway. Later, mitochondrial permeabilization and subsequently release of cytochrome *c* could initiate the activation cascade of caspases. The cleavage of caspase-3 promotes DNA fragmentation and leads to apoptotic cell dismantling (48). JC-1, a cationic carbocyanine dye, detects ΔΨm and can be used to assess the state of the mitochondrial polarization in healthy and apoptotic cells (49). As shown in Fig. 5A, uninfected AAE2 cells showed healthy mitochondria containing polarized inner membranes and exhibited orange fluorescence (colocalized red and green fluorescence). In contrast, the *Rp*-infected cells exhibited a significantly reduced orange fluorescence and remarkable increased green fluorescence. Mitochondrial depolarization was also indicated by a significant reduction in the red/green fluorescence intensity ratio in *Rp*-infected cells and carbonyl cyanide 3-chlorophenylhydrazone (CCCP; positive control) treated cells (Fig. 5B) relative to uninfected AAE2 cells. A similar effect was seen in *Rp*-infected ISE6 cells (Fig. S4); i.e., more green fluorescence and a reduction of the red/green fluorescence intensity ratio were observed.

10.1128/mSystems.01209-20.6 FIG S4 ISE6 cell mitochondrial membrane potential observed in staining with MitoPT JC-1. (A) Uninfected ISE6 cells (left); *Rp*-infected ISE6 cells (right). (B) Red and green fluorescence signal in uninfected ISE6 cells (control) and *Rp*-infected cells was measured using a Synergy H1 plate reader. The ratio of fluorescence signals was calculated and analyzed by Student’s two-tailed *t* test. Download FIG S4, TIF file, 0.4 MB. Copyright © 2021 Wang et al. 2021 Wang et al. https://creativecommons.org/licenses/by/4.0/ This content is distributed under the terms of the Creative Commons Attribution 4.0 International license.

We investigated further whether cytochrome *c* was released from mitochondria into the cytosol after infection with *Rp* at 72 h p.i. in AAE2 cells. Pearson’s correlation coefficient (PCC) and overlap coefficient determined according to Manders’ calculations (MOC) (Fig. 5C) were analyzed following established guidelines (50, 51). Although the PCC value remained unchanged, a decreasing MOC value demonstrated that the degree of colocalization of cytochrome *c* and mitochondria declined, suggesting that *Rp* infection induced cytochrome *c* release into the cytosol. As expected, in uninfected AAE2 cells (Fig. 5D, top row), mitochondria (Fig. 5D, panel 1, red signal) colocalized with FITC-labeled cytochrome *c* (Fig. 5D, panel 2, green signal), resulting in yellow fluorescence in the merged (Fig. 5D, panel 3, blue signal; DAPI-stained nuclei) and enlarged (Fig. 5D, panel 4, yellow signal) images. This suggested a higher colocalization level than in infected AAE2 cells (Fig. 5D, bottom row), indicating a lack of cytochrome *c* release to the cytosol. Meanwhile, *Rp*-infected AAE2 cells also exhibited higher levels of caspase-3/7 enzyme activity than uninfected cells, reflecting the activation of the caspase cascade (Fig. 5E and F). These results strongly suggested that the intrinsic apoptosis pathway is activated in tick cells in response to *Rp* infection.

### Apoptosis induction requires *Rp* intracellular replication.

**FIG 6:**
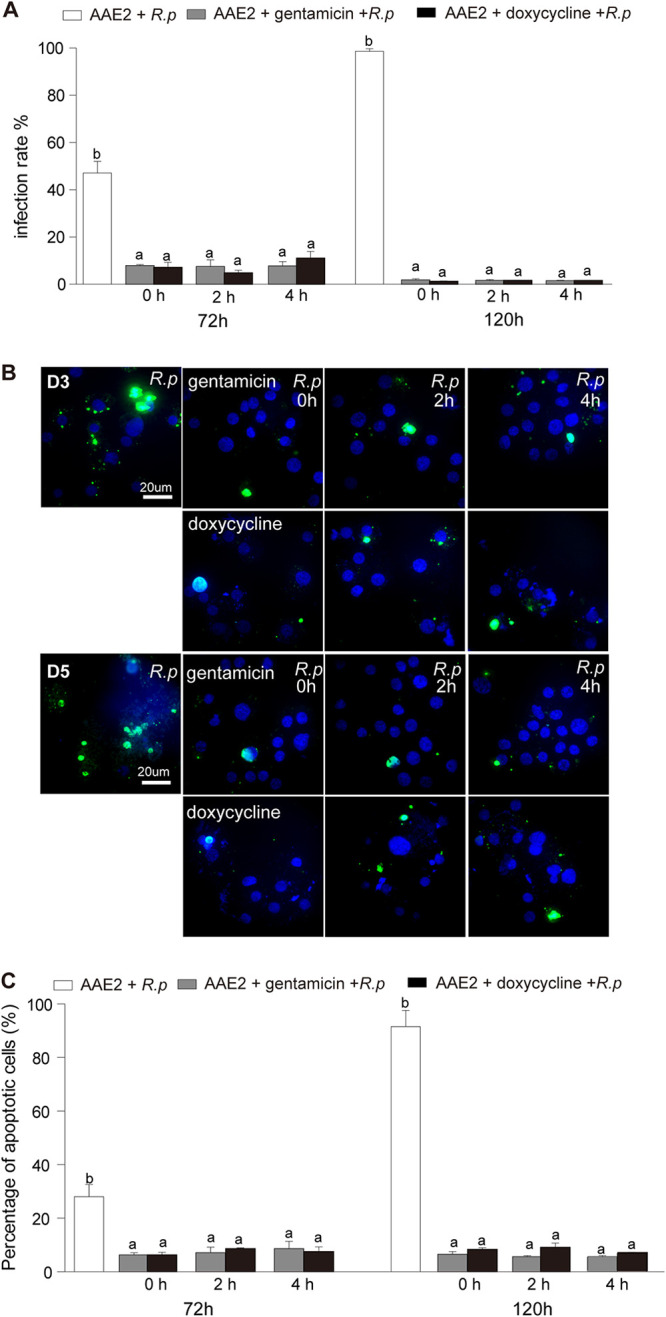
Effects of antibiotics on apoptosis activation and *Rp* replication. AAE2 cells were infected with host cell-free *Rp* for 0, 2, and 4 h, then treated with gentamicin or doxycycline, and collected at 72 h and 120 h for analysis. (A) *Rp* infection rate identified with Giemsa staining. (B) *Rp*-infected AAE2 cells were fixed and labeled with TUNEL (green). Bar, 20 μm. Blue DAPI staining corresponds to the nuclei. (C) Percent apoptotic cells (number of TUNEL-positive cells/number of DAPI-positive cells) in different fields. In panels A and C, data are means ± SD, and different letters above the columns indicate significant differences (ANOVA, followed by Bonferroni test).

Chlamydia psittaci, an obligately intracellular organism, induces apoptosis at a later stage of infection in an intracellular replication-dependent manner (44). To investigate whether *Rp* intracellular replication is required for apoptosis induction, we used two types of antibiotics (doxycycline, which inhibits intracellular rickettsial growth, and gentamicin, which kills extracellular rickettsiae) (52) to treat infected AAE2 cells. AAE2 cells were incubated with host cell-free *Rp* at 4°C for 2 h to achieve synchronized infections, and cold exposure did not generate extra stress to induce apoptosis in infected AAE2 cells (Fig. S5). Treatment with gentamicin or doxycycline at early time points after infection (0, 2, and 4 h) significantly decreased the *Rp* infection rate measured at 72 h and 120 h p.i. relative to untreated cells (Fig. 6A). The percentage of apoptotic cells was reduced sharply upon gentamicin or doxycycline treatment compared to that in untreated cells (Fig. 6C) by TUNEL assay (Fig. 6B) but was similar to that in uninfected cells (date not shown). Interestingly, there was no significant difference among 0-, 2-, and 4-h-postinfection time groups regarding the apoptotic rate, perhaps suggesting that apoptosis induction might occur after replication commences. Meanwhile, the quantities of *Rp* were significantly higher in untreated cells than in gentamicin- or doxycycline-treated cells. The significant differences in the quantities of *Rp*, which were higher in gentamicin-treated cells than doxycycline-treated cells, reflected the differences between the antibiotic mechanisms of gentamicin and doxycycline (Fig. S6). Mitochondrial membrane potential changes are also indicated by a significant increase in the red/green fluorescence intensity ratio (Fig. S7) relative to untreated AAE2 cells, suggesting that antibiotic treatments reduced *Rp* replication and therefore apoptosis induction. These results indicated that *Rp* intracellular replication was required for apoptosis induction, while apoptosis facilitated *Rp* replication.

10.1128/mSystems.01209-20.7 FIG S5 Cold (4°C, 2 h) treatment to synchronize *Rp* infection did not directly activate apoptosis in AAE2 cells. (A) AAE2 cells were exposed to pRAM18dSFA-transformed *Rp* at 4°C or 34°C (control, no cold treatment), and cells were fixed and labeled with TUNEL (green). Bar, 20 μm. Blue DAPI staining corresponds to the nuclei. (B) Percent apoptotic cells (number of TUNEL-positive cells/number of DAPI-positive cells) in different fields. Data are means and SD, and different *P* values above the columns indicate significance of the differences. Download FIG S5, TIF file, 0.8 MB. Copyright © 2021 Wang et al. 2021 Wang et al. https://creativecommons.org/licenses/by/4.0/ This content is distributed under the terms of the Creative Commons Attribution 4.0 International license.

10.1128/mSystems.01209-20.8 FIG S6 The quantities of *Rp* in AAE2 cell cultures after antibiotic treatment. *Rp* genomic DNA was isolated from AAE2 cell cultures (per milliliter) and measured by qPCR using *gltA* as a reference. Data are means and SD, and different letters above the columns indicate significant differences. Download FIG S6, TIF file, 0.9 MB. Copyright © 2021 Wang et al. 2021 Wang et al. https://creativecommons.org/licenses/by/4.0/ This content is distributed under the terms of the Creative Commons Attribution 4.0 International license.

10.1128/mSystems.01209-20.9 FIG S7 Mitochondrial membrane potential of *Rp*-infected AAE2 cells after antibiotic treatment. Red and green fluorescence signals in *Rp*-infected AAE2 cells treated or not with gentamicin or doxycycline for the times indicated was measured using a Synergy H1 plate reader. The ratios of red/green fluorescence were calculated; data are means and SD, and different letters next to the columns indicate significant differences. Download FIG S7, TIF file, 0.3 MB. Copyright © 2021 Wang et al. 2021 Wang et al. https://creativecommons.org/licenses/by/4.0/ This content is distributed under the terms of the Creative Commons Attribution 4.0 International license.

### Apoptosis is not observed in mammalian cells during later stages of *Rp* infection.

**FIG 7:**
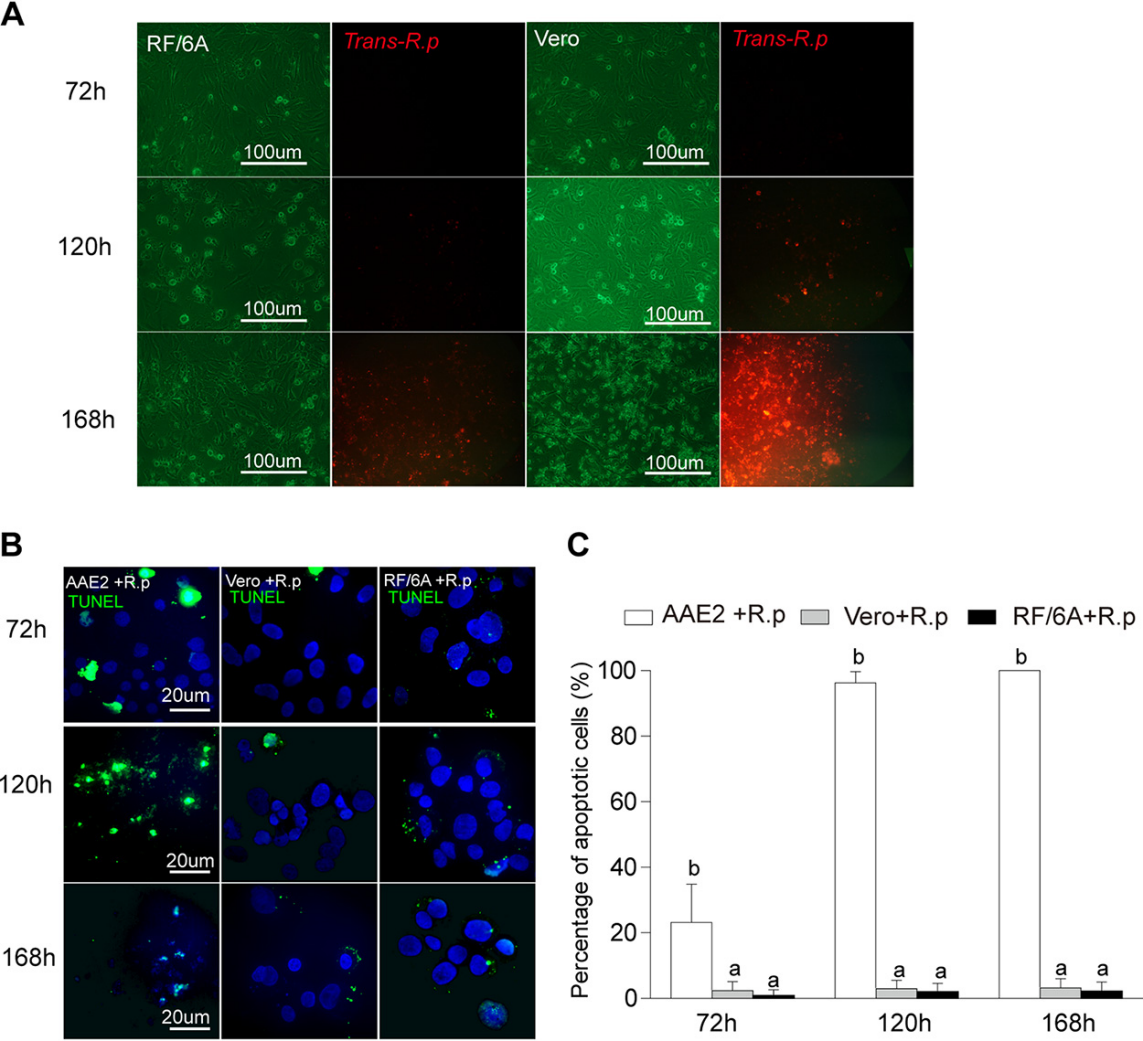
No later stage of apoptosis is observed in RF/6A and Vero cells after *Rp* infection. (A) RF/6A and Vero cells were infected with host cell-free pRAM18dSFA-transformed *Rp* (72, 120, and 168 h) and observed under a fluorescence microscope for infection and replication. Bar, 100 μm. (B) *Rp*-infected AAE2, RF/6A, and Vero cells were fixed and labeled with TUNEL (green). Bar, 20 μm. Blue DAPI staining corresponds to the nuclei. (C) Percent apoptotic cells (number of TUNEL-positive cells/number of DAPI-positive cells) in different fields. In panel C, data are means and SD, and different letters above the columns indicate significant differences (ANOVA, followed by Bonferroni test).

A previous study indicated that R. rickettsii protects infected vascular endothelial cells from apoptosis through the activation of the NF-κB signaling pathway (24). To discern the difference in apoptosis response after *Rp* infection between mammalian and tick cells, we investigated whether apoptosis was induced after *Rp* infection in Vero cells (a standard cell line used in rickettsiology) and RF/6A cells (monkey endothelial cells; SFG members mainly infect mammalian endothelial cells) compared with AAE2 cells, using plasmid-transformed *Rp* expressing a red fluorescent protein (53). Even though transformed *Rp* replicated quickly, resulting in high rates of infection after 72 h in Vero and RF/6A cells (Fig. 7A), there was no significant change in the number of apoptotic cells at any stage of *Rp* infection relative to that in uninfected cells (Fig. S8). In contrast, the percentage of apoptotic cells was remarkably lower than that in *Rp*-infected AAE2 cells (Fig. 7B and C). The apoptosis response is distinctly different in tick and mammalian cells, suggesting that *Rp* might utilize two different survival strategies to modulate apoptosis in arthropod vectors and mammalian hosts.

10.1128/mSystems.01209-20.10 FIG S8 No apoptotic response in uninfected Vero and RF/6A cells (negative controls). Cells were fixed and labeled with TUNEL (green). Bar, 20 μm. Blue DAPI staining corresponds to the nuclei. Download FIG S8, TIF file, 1.8 MB. Copyright © 2021 Wang et al. 2021 Wang et al. https://creativecommons.org/licenses/by/4.0/ This content is distributed under the terms of the Creative Commons Attribution 4.0 International license.

### Human pathogenicity of spotted fever group rickettsiae is not linked to apoptosis activation.

**FIG 8:**
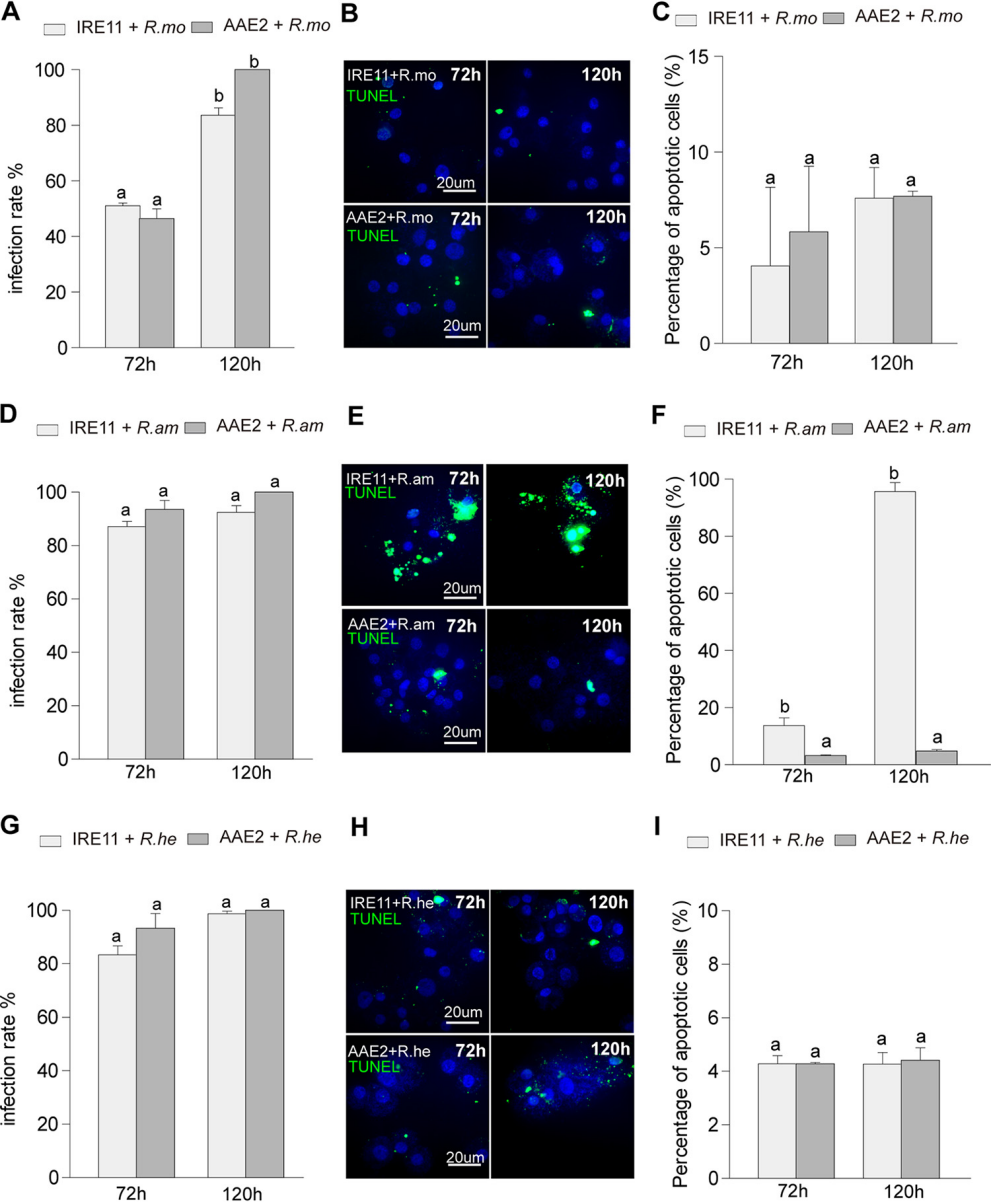
Mammalian pathogenicity is not required for apoptosis activation among SFG species. IRE11 or AAE2 cells were infected with host cell-free *R. monacensis*, *R. amblyommatis*, and *R. helvetica* and then collected at 72 h and 120 h for analysis. (A to C) *R. monacensis* infection rates were identified with Giemsa staining (A). (B) *R. monacensis*-infected AAE2 or IRE11 cells were fixed and labeled with TUNEL (green). Bar, 20 μm. Blue DAPI staining corresponds to the nuclei. (C) Percent apoptotic cells (number of TUNEL-positive cells/number of DAPI-positive cells) in different fields after *R. monacensis* infection. (D to F) *R. amblyommatis* infection rates were identified with Giemsa staining (D). (E) *R. amblyommatis*-infected AAE2 or IRE11 cells were fixed and labeled with TUNEL (green). Bar, 20 μm. Blue DAPI staining corresponds to the nuclei. (F) Percent apoptotic cells (number of TUNEL-positive cells/number of DAPI-positive cells) in different fields after *R. amblyommatis* infection. (G to I) *R. helvetica* infection rates were identified with Giemsa staining (G). (H) *R. helvetica*-infected AAE2 or IRE11 cells were fixed and labeled with TUNEL (green). Bar, 20 μm. Blue DAPI staining corresponds to the nuclei. (I) Percent apoptotic cells (number of TUNEL-positive cells/number of DAPI-positive cells) in different fields after *R. helvetica* infection. In panels A, C, D, F, G, and I, data are means and SD, and different letters above the columns indicate significant differences.

To examine whether a correlation exists between apoptosis induction and rickettsial pathogenicity with different *Rickettsia* spp., we tested apoptosis responses in tick vector cells to three other spotted fever group rickettsiae that differed in pathogenicity for humans: Rickettsia monacensis IrR/Munich and Rickettsia helvetica strain C9P9, which are both associated with the European sheep tick (Ixodes ricinus), the first being of uncertain pathogenicity and the second a human pathogen, and Rickettsia amblyommatis AaR/SC, a nonpathogenic species symbiotically associated with the lone star tick, *Amblyomma americanum*. At the same infection stage (72 h and 120 h p.i.), there was no significant difference in the infection rates of IRE11 and AAE2 cells between the three rickettsial species (Fig. 8A, D, and G). However, the apoptosis responses of the tick cells to the three rickettsial species were distinctly different. Neither *R. monacensis* (Fig. 8B and C) nor *R. helvetica* (Fig. 8H and I) infection triggered a significant increase in apoptotic rate in IRE11 or AAE2 cells, unlike our previous observations for *Rp* (Fig. 2; Fig. S3). *R. amblyommatis* did not induce apoptosis in AAE2 cells but exhibited apoptosis activation in IRE11 cells, as indicated by TUNEL assay (Fig. 8E and F). Together, these results suggested no direct correlation between apoptosis activation and rickettsial pathogenicity in SFG rickettsiae.

## DISCUSSION

Intracellular bacteria have to constantly battle with the host and vector for survival and replication, and they have developed complex and highly efficient strategies to overcome innate and adaptive immune responses (54). As part of the innate immune response, apoptosis plays an essential role in regulating intracellular bacterial invasion and survival (55). As obligately intracellular parasites, rickettsiae have evolved a variety of strategies modulating host apoptosis by activating survival pathways and directly regulating cytokine synthesis, blocking caspase activation, and upregulating antiapoptotic molecular signals (22–24, 30, 55). Despite the widespread recognition of the importance of apoptosis in host-pathogen interactions, little is known regarding whether and how rickettsiae modulate apoptosis of their arthropod vectors for their own survival and replication. This study shows that infection of tick cells by SFG rickettsiae significantly activates the apoptosis pathway and that the activation of apoptosis facilitates rickettsial infection and replication. We further reveal that the mitochondrion-dependent apoptosis activated by the rickettsiae is likely to rely on intracellular replication. Furthermore, we show that there is no direct correlation between rickettsia pathogenicity and apoptosis activation in tick vectors.

The ability of intracellular bacteria to fine-tune the balance between anti-and proapoptotic roles has been revealed in *Chlamydia* and *Mycobacterium* (43, 44, 56, 57). Similarly, *Rp* exhibited both anti- and proapoptotic roles during different stages of tick cell infection (Fig. 2). The early stage of infection demonstrated antiapoptotic activity, perhaps suggesting a protective role in maintaining the metabolic balance of the infected cells, as shown previously for *Chlamydia* and *Mycobacterium* (43, 57). Because *Rp* intracellular replication was required for proapoptotic activity, subsequent activation of apoptosis at the later stage of infection might promote rickettsial cell-to-cell spread. Different cell lines from different tick species have different morphologic characteristics and ontogenies (58). Interestingly, *Rp*-induced apoptosis is independent of cell type, suggesting that apoptosis is a fundamentally conserved response in tick vectors. Therefore, revealing homologies in the apoptosis machinery of diverse tick species and evaluating their evolution trajectories will provide more valuable explanations for tick evolutionary biology.

Mitochondrial membrane permeabilization and cytochrome *c* release into the cytosol after *Rp* infection reflected apoptosis activation by the intrinsic pathway (Fig. 5). Although mitochondrion-dependent apoptotic pathways are well-known in arthropods, the role of cytochrome *c* has been considered controversial. For example, cytochrome *c* failed to activate *Drosophila* caspase and was not involved in apoptosis (59), whereas it was required for Spodoptera frugiperda, Spodoptera litura, and Lymantria dispar apoptosis (60–63). We found that tick cell apoptosis activated by *Rp* allowed leakage of cytochrome *c* into the cytosol accompanied by its decline in the mitochondria. However, another rickettsial agent, A. phagocytophilum, manipulates multiple defense systems of its tick vector in specific tissues at different times to ensure successful acquisition and transmission by upregulating the JAK/STAT pathway in tick midguts and also preventing the release of cytochrome *c* in salivary glands (30). This appears to be a conserved mechanism, as A. phagocytophilum utilizes several mechanisms to inhibit apoptosis in tick cells derived from different species, such as targeting the JAK/STAT pathway and decreasing FAS expression, interfering with the endoplasmic reticulum (ER) and glucose metabolism, and changing molecules directly to induce mitochondrial stress (55). Unlike the *Anaplasmataceae*, the *Rickettsiaceae* are not confined in an intracellular compartment and are in direct contact with host cell cytoplasm. We speculate that these differences may contribute to the different tick cell responses to pathogens they transmit, but much more research is needed to fully explain how apoptosis is modulated, as different SFG rickettsia species or even strains within the same species caused different cellular responses (64; this paper). Undoubtedly, the growing number of available rickettsial genome sequences will help to improve understanding how diverse rickettsiae prompt different responses from vectors and hosts.

The apoptosis regulatory molecules (ARMs) produced by intracellular bacteria, including secreted exoproducts (e.g., exotoxins) and nonsecreted endoproducts (e.g., structural moieties and lipoteichoic acid), could target host cells and trigger apoptosis pathways through multiple molecular mechanisms (65). However, little is known regarding how rickettsial ARMs manipulate apoptosis in tick vectors. As far as we are aware, our study is the first attempt to examine the key factors to trigger apoptosis activation by an SFG rickettsia in its tick vector. Two antibiotics, gentamicin (which inhibits rickettsiae while they are extracellular) and doxycycline (which can act on intracellular rickettsiae), significantly decreased the rate of apoptosis in infected tick cells, suggesting that rickettsial intercellular replication is required for apoptosis activation (Fig. 6), as previously seen in C. psittaci (44). However, rickettsial intercellular replication does not activate apoptosis in mammalian host cells (Fig. 7), reflecting another strategy in modulating apoptosis response, possibly similar to R. rickettsii (24). Much progress has been made in understanding how rickettsiae survive and replicate in host cells, including the role particular bacterial effectors play to manipulate host cells by rearranging the cytoskeleton, polymerizing host actin, altering host phagosome trafficking, and forming a membrane-bound compartment (54, 66). In the future, it will be important to identify novel factors and effectors that participate in manipulating host apoptosis and to determine whether they function in a rickettsia-specific manner or induce globally conserved responses in their arthropod vector.

The explosion of studies focused on bacterium-induced apoptosis and diverse apoptotic response mechanisms of host cells highlights the critical role apoptosis plays in bacterial pathogenesis and antibacterial immune response (67). Members of the genus *Rickettsia* exhibit a range of virulence characteristics, from harmless endosymbionts harbored by invertebrates and protozoa to the causative agents of severe disease (4). By comparing the apoptosis responses of tick cells to rickettsiae of uncertain pathogenicity (*R. monacensis*), a human pathogen (*R. helvetica*), and a nonpathogenic symbiont (*R. amblyommatis*), we found no direct correlation between rickettsial pathogenicity and apoptosis activation. Nevertheless, the severe human pathogen R. rickettsii has been reported either to have little effect on survival of its tick vectors or to kill them (68–71), possibly depending on the strain examined. Obviously, this finding further demonstrates that rickettsiae might utilize two different survival strategies to modulate apoptosis in arthropod vectors and mammalian hosts (Fig. 7). The opposite apoptosis response to *R. amblyommatis*, i.e., induction of apoptosis in its natural vector tick-derived cell lines while inhibiting apoptosis in cells from other tick species, may indicate that the endosymbiont may present a way to control the natural vector.

Here, we investigated the mechanisms of apoptosis activation by SFG rickettsiae in tick vector cells and comprehensively analyzed how apoptosis regulates SFG rickettsia survival in tick versus vertebrate hosts. Our findings also provide evidence that *Rp*, an SFG rickettsia, triggers mitochondrion-dependent apoptosis in its tick vector cells, promoting rickettsial infection and replication in the vector cells. Intracellular replication is required for triggering apoptosis; however, whether toxins or virulence factors produced by *Rp* during replication play a role in activating apoptosis remains to be determined.

During their life cycle in nature, rickettsiae must overcome a more complex system of innate barriers to infection and persistence in ticks than *in vitro*. These include structural features, such as the peritrophic matrix that separates the infectious blood meal from gut epithelium (72), and the immune responses of specific target tissues in different species of ticks reacting to a broad range of *Rickettsia* species. For instance, how does *Rp* “tinker” with the tick’s apoptosis pathway to infect or to suppress or regulate immune responses to its own benefit? Is induction of apoptosis associated with specific tissues (salivary glands, gut, and reproductive systems), and what are the essential tick immunity factors that induce apoptosis? The knowledge gained from studies addressing these questions will improve our understanding of the modulation of apoptosis in arthropod vectors by rickettsiae. Although apoptosis mechanisms have been investigated in the order *Rickettsiales* at an accelerating pace, especially in the family *Anaplasmataceae*, what is currently understood about apoptosis induced by the family *Rickettsiaceae* is merely the tip of the iceberg. Findings from this study fill a critical void in our understanding of vector-rickettsia-host molecular interactions and provide valuable clues for designing new strategies to block the transmission of arthropod-vectored pathogens, particularly the family *Rickettsiaceae*.

## MATERIALS AND METHODS

### Cell cultures.

All tick cells (cell lines ISE6, IRE11, BME26, and AAE2) were cultured at 34°C in complete medium (L-15C300, 5% fetal bovine serum [FBS], 5% tryptose phosphate broth, and 0.1% lipoprotein concentrate) as reported previously (73). RF/6A (a choroid-retina endothelial cell line) and Vero cells were cultured at 34°C in complete RPMI 1640 medium with 10% FBS.

### *Rickettsia* strains, growth, and purification.

All *Rickettsia* strains (listed in Table S1) were added to tick cell cultures in complete medium with 10% FBS and additionally supplemented with NaHCO_3_ and HEPES buffer as described elsewhere (74). Infected cells were transferred to 2.0-ml Eppendorf tubes containing 60/90 grit silicon carbide, vortexed at maximum speed for 30 s, and purified by passage through a Whatman 2.0-μm filter. Host cell-free rickettsiae were was collected by centrifugation at 13,000 × *g* for 5 min at 4°C and resuspended in complete medium. Viable rickettsiae were counted using a Petroff-Hausser chamber, as previously described (74). Culture flasks (25 cm^2^) were inoculated with *Rp* at an MOI of 1.

10.1128/mSystems.01209-20.1 TABLE S1 SFG *Rickettsia* species and strains used in this study. Download Table S1, DOCX file, 0.01 MB. Copyright © 2021 Wang et al. 2021 Wang et al. https://creativecommons.org/licenses/by/4.0/ This content is distributed under the terms of the Creative Commons Attribution 4.0 International license.

### RNA isolation and qRT-PCR.

Total RNA was isolated from cells using TRI reagent (Sigma) and purified using an RNA Clean & Concentrator kit (Zymo Research). The quantity and quality of RNA were evaluated using a DS-11 series spectrophotometer/fluorometer (DeNovix). cDNA was synthesized using the SYBR PrimeScript reverse transcription-PCR (RT-PCR) kit II (TaKaRa). The expression levels of apoptosis-related genes in different treatments were determined using qPCR on the Mx3005P real-time system (Stratagene) with SYBR green detection (Agilent Technologies). Primers used in this study are listed in Table S2.

10.1128/mSystems.01209-20.2 TABLE S2 qRT-PCR primers. Download Table S2, DOCX file, 0.01 MB. Copyright © 2021 Wang et al. 2021 Wang et al. https://creativecommons.org/licenses/by/4.0/ This content is distributed under the terms of the Creative Commons Attribution 4.0 International license.

### DNA extraction and laddering gel.

Total DNA was extracted from infected cells using the Gentra Puregene cell kit (Qiagen), following the manufacturer's instructions for DNA purification from cultured cells. The purified DNA was eluted with Tris-EDTA buffer, and quantity and quality were evaluated using a DS-11 series spectrophotometer/fluorometer (DeNovix). DNA was electrophoretically separated on 1.2% agarose gels.

### *Rickettsia* DNA extraction and real-time PCR.

Genomic DNA was extracted from *Rp*-infected cells using the Puregene Core A kit (Qiagen, Valencia, CA) according to the manufacturer's protocol for Gram-negative bacteria. The quantity and quality of purified genomic DNA were evaluated with a DS-11 series spectrophotometer/fluorometer (DeNovix), and qPCR was used to estimate the copy number of the single-copy rickettsial citrate synthase (*gltA*) gene as previously described (53). Primers are listed in Table S2 (75).

### Annexin V/PI staining.

The early versus later stage of apoptosis was analyzed using an annexin V-FITC apoptosis staining/detection kit (Abcam). Wild-type *Rp* organisms were used to infect AAE2 cells for 4 days, and uninfected cells served as controls. Cells were collected by centrifugation (500 × *g* for 5 min), resuspended in 1× binding buffer, and incubated with FITC-conjugated annexin V, PI, and NucBlue live cell stain (ReadyProbes reagent; Thermo Fisher Scientific) in the dark for 15 min at room temperature. The cells were then deposited onto microscope slides (Cytospin centrifuge; Thermo Fisher), and imaged on an Olympus BX61 DSU confocal microscope fitted with a 60× objective. Cells were observed using a multiwavelength filter (4′,6-diamidino-2-phenylindole [DAPI], excitation at 365 nm and emission at 480 nm; FITC, excitation at 495 nm and emission at 519 nm; mCherry, excitation at 550 to 590 nm and emission at 550 to 650 nm). All treatments were replicated three times.

### TUNEL assay.

Apoptotic cell death was analyzed using the *in situ* cell death detection kit (Roche). Cells were immobilized onto slides, fixed in 4% paraformaldehyde for 1 h at room temperature, permeabilized with 0.1% Tween 20 (in phosphate-buffered saline [PBS]), and then incubated with the TUNEL reagents (TdT enzyme-dUTP, 1:10) for 1 h at 37°C. The slides were mounted in Fluoroshield mounting medium with DAPI (Vector Laboratories) and analyzed under an Olympus BX61 DSU confocal microscope with a 60× objective. Dual fluorescence properties were observed using a multiwavelength filter as described above. All treatments were replicated three times.

### Mitochondrial membrane depolarization detection by JC-1.

Loss of mitochondrial ΔΨm was detected using a cationic fluorescent redistribution dye, 5,5′,6,6′-tetrachloro-1,1′,3,3′-tetraethylbenzimidazolocarbocyanine iodide (JC-1; Immunochemistry) following the manufacturer’s instructions. Host cell-free *Rp* (MOI = 1) organisms were used to infect cells for 72 h; uninfected cells served as negative controls, and uninfected cells with CCCP served as the positive control. All samples were incubated with JC-1 dye and analyzed for a shift in emission from red (∼590 nm) to green (∼529 nm) using a fluorescence plate reader (Biotek M3). JC-1-stained cells were immobilized onto slides and analyzed under an Olympus BX61 DSU confocal microscope using a 60× objective. Dual fluorescence properties were observed using a multiwavelength filter as described above. All treatments were replicated three times.

### Caspase 3/7 enzyme activity assay.

Caspase enzyme activity was detected using the Magic Red caspase-3/7 assay kit (Immunochemistry), following the manufacturer's instructions. Host cell-free *Rp* (MOI = 1) organisms were used to infect cells for 72 h, and uninfected cells served as controls. All samples were incubated with Magic Red substrate and then immobilized onto slides. Specimens were mounted in Fluoroshield mounting medium with DAPI and imaged under an Olympus BX61 DSU confocal microscope using a 60× objective. Dual-fluorescence properties were observed using a multiwavelength filter as described above. All treatments were replicated three times.

### Colocalization assay.

Host cell-free *Rp* (MOI = 1) organisms were used to infect cells for 72 h, and uninfected cells served as controls. Cells were incubated with 50 μM MitoTracker deep red, immobilized onto slides, and fixed in 4% paraformaldehyde for 1 h at room temperature. They were then permeabilized with 0.1% Tween 20 (in PBS), incubated with FITC-conjugated antibody to cytochrome *c*, mounted in Fluoroshield mounting medium with DAPI, and imaged under a Nikon A1si spectral confocal microscope using a 60× objective. Triple-fluorescence properties were observed using a multiwavelength filter (DAPI; FITC; Cy5, excitation at 647 nm and emission at 665 nm). Images were processed using the program NIS-Elements View 4.50 (University Imaging Centers at the University of Minnesota, Twin Cities, MN). All treatments were replicated 3 times. The colocalization of cytochrome *c* in mitochondria was assessed qualitatively and quantified by determining the degree of the area of two signals overlapping, according to Pearson’s coefficient and overlap coefficient according to Manders’ calculations by Image Fiji (JaCoP plugin and Colocalization Threshold plugin).

### Monitoring time course of apoptosis activation in response to *Rp* infection.

Host cell-free *Rp* (MOI = 1) organisms were used to infect AAE2 cells, and then the infected samples were collected at 6 different time points (from 0 h to 120 h); uninfected cells served as controls. The replication dynamics of *Rp* was examined by Giemsa staining. The *Rp* numbers were measured by qPCR compared with citrate synthase (*gltA*) gene copies. TUNEL was used to analyze the time course of apoptosis response to *Rp* infection at different time points. All treatments were replicated three times.

### Treatment with apoptosis inhibitors and inducers.

Z-VAD-FMK (30 μM), sabutoclax (50 μM), PAC-1 (100 μM), and etoposide (100 μM) working solutions were prepared in DMSO, diluted in 1× PBS, and then used separately to treat AAE2 cells. Next, host cell-free *Rp* (MOI = 1) organisms were used to infect the inducer- or inhibitor-treated cells, and the samples were collected at 72 h and 120 h, with untreated cells serving as controls. The infection rate (number of *Rp*-infected cells/number of total cells) was identified by Giemsa staining (images not shown), while the quantities of *Rp* organisms were determined by qPCR in comparison with citrate synthase (*gltA*) gene copies. TUNEL was used to detect apoptotic cells in cultures treated with the respective inhibitors or inducers. All treatments were replicated three times.

### Treatment with antibiotics.

Host cell-free *Rp* (MOI = 1) organisms were incubated with AAE2 cells for 0, 2, and 4 h at 4°C, and residual, nonadherent rickettsiae were washed away by centrifugation. Gentamicin (16.7 μg/ml) and doxycycline (20 μg/ml) working solutions were prepared in water, diluted in 1× PBS, and used separately to treat rickettsia-challenged cells for 0, 2, and 4 h. Cultures were sampled at 72 h and 120 h, with uninfected and untreated cells serving as controls. The infection rate was monitored by Giemsa staining (images not shown), while the quantities of *Rp* were determined by qPCR compared with *gltA* gene copies. The effects of antibiotics on apoptosis were examined by carrying out TUNEL and analyzing mitochondrial membrane depolarization. All treatments were replicated three times.

### Monitoring the time course of apoptosis activation in response to *Rp* infection in mammalian cells.

Host cell-free pRAM18dSFA-transformed *Rp* (MOI = 1) organisms were used to infect Vero and RF/6A cells. After 72 h, 120 h, and 168 h, infected samples were collected and imaged under a Nikon Diaphot inverted phase-contrast microscope fitted for fluorescence microscopy; uninfected cells served as controls. To discern the difference in apoptosis response after *Rp* infection between mammalian and tick cells, host cell-free *Rp* (MOI = 1) organisms were used to infect AAE2, Vero, and RF/6A cells. Infected samples were then collected at 72 h, 120 h, and 168 h, and uninfected cells served as controls. TUNEL was used to detect apoptosis in different cell lines. All treatments were replicated three times.

### Monitoring the time course of apoptosis activation in response to *Rickettsia* sp. infection.

Host cell-free *R. monacensis*, *R. amblyommatis*, and *R. helvetica* (MOI = 1) were separately used to infect AAE2 and IRE11 cells. The infected samples were then collected at 72 h and 120 h; uninfected cells served as the controls. The infection rate was monitored by Giemsa staining (images not shown). TUNEL was used to detect apoptosis induced by the different *Rickettsia* spp. All treatments were replicated three times.

### Statistical analysis.

We applied one-way analysis of variance (ANOVA) for analysis of all data on gene expression, percentages of apoptotic cells, the quantities of *Rp* DNA, infection rate, the ratio of red/green fluorescence, and the colocalization coefficient. This was followed by Bonferroni analysis when there were ≥3 treatments. Student’s two-tailed *t* test was applied when there were only two treatments. Differences were judged significant when the *P* value was <0.05.

## References

[1] Raoult D, Roux V. 1997. Rickettsioses as paradigms of new or emerging infectious diseases. Clin Microbiol Rev 10:694–719. doi:10.1128/CMR.10.4.694-719.1997. 9336669 PMC172941

[2] Tomassone L, Portillo A, Nováková M, De Sousa R, Oteo JA. 2018. Neglected aspects of tick-borne rickettsioses. Parasit Vectors 11:263. doi:10.1186/s13071-018-2856-y. 29690900 PMC5937841

[3] Gillespie JJ, Williams K, Shukla M, Snyder EE, Nordberg EK, Ceraul SM, Dharmanolla C, Rainey D, Soneja J, Shallom JM, Vishnubhat ND, Wattam R, Purkayastha A, Czar M, Crasta O, Setubal JC, Azad AF, Sobral BS. 2008. Rickettsia phylogenomics: unwinding the intricacies of obligate intracellular life. PLoS One 3:e2018. doi:10.1371/journal.pone.0002018. 19194535 PMC2635572

[4] Murray GG, Weinert LA, Rhule EL, Welch JJ. 2016. The phylogeny of Rickettsia using different evolutionary signatures: how tree-like is bacterial evolution? Syst Biol 65:265–279. doi:10.1093/sysbio/syv084. 26559010 PMC4748751

[5] Parola P, Paddock CD, Raoult D. 2005. Tick-borne rickettsioses around the world: emerging diseases challenging old concepts. Clin Microbiol Rev 18:719–756. doi:10.1128/CMR.18.4.719-756.2005. 16223955 PMC1265907

[6] Van Kirk LS, Hayes SF, Heinzen RA. 2000. Ultrastructure of *Rickettsia rickettsii* actin tails and localization of cytoskeletal proteins. Infect Immun 68:4706–4713. doi:10.1128/iai.68.8.4706-4713.2000. 10899876 PMC98416

[7] Joshi SG, Francis CW, Silverman DJ, Sahni SK. 2003. Nuclear factor κB protects against host cell apoptosis during *Rickettsia rickettsii* infection by inhibiting activation of apical and effector caspases and maintaining mitochondrial integrity. Infect Immun 71:4127–4136. doi:10.1128/iai.71.7.4127-4136.2003. 12819104 PMC162038

[8] Chan YGY, Riley SP, Martinez JJ. 2010. Adherence to and invasion of host cells by spotted fever group Rickettsia species. Front Microbiol 1:139. doi:10.3389/fmicb.2010.00139. 21687751 PMC3109342

[9] Kleba B, Clark TR, Lutter EI, Ellison DW, Hackstadt T. 2010. Disruption of the *Rickettsia rickettsii* Sca2 autotransporter inhibits actin-based motility. Infect Immun 78:2240–2247. doi:10.1128/IAI.00100-10. 20194597 PMC2863521

[10] Elmore S. 2007. Apoptosis: a review of programmed cell death. Toxicol Pathol 35:495–516. doi:10.1080/01926230701320337. 17562483 PMC2117903

[11] Taatjes DJ, Sobel BE, Budd RC. 2008. Morphological and cytochemical determination of cell death by apoptosis. Histochem Cell Biol 129:33–43. doi:10.1007/s00418-007-0356-9. 18000678 PMC2137940

[12] Ashida H, Mimuro H, Ogawa M, Kobayashi T, Sanada T, Kim M, Sasakawa C. 2011. Cell death and infection: a double-edged sword for host and pathogen survival. J Cell Biol 195:931–942. doi:10.1083/jcb.201108081. 22123830 PMC3241725

[13] Hilbi H, Moss JE, Hersh D, Chen Y, Arondel J, Banerjee S, Flavell RA, Yuan J, Sansonetti PJ, Zychlinsky A. 1998. *Shigella*-induced apoptosis is dependent on caspase-1 which binds to IpaB. J Biol Chem 273:32895–32900. doi:10.1074/jbc.273.49.32895. 9830039

[14] Sansonetti PJ, Arondel J, Cavaillon JM, Huerre M. 1995. Role of interleukin-1 in the pathogenesis of experimental shigellosis. J Clin Invest 96:884–892. doi:10.1172/JCI118135. 7635983 PMC185275

[15] Gao LY, Kwaik YA. 2000. The modulation of host cell apoptosis by intracellular bacterial pathogens. Trends Microbiol 8:306–313. doi:10.1016/s0966-842x(00)01784-4. 10878765

[16] Massari P, Ho Y, Wetzler LM. 2000. *Neisseria meningitidis* porin PorB interacts with mitochondria and protects cells from apoptosis. Proc Natl Acad Sci U S A 97:9070–9075. doi:10.1073/pnas.97.16.9070. 10922061 PMC16823

[17] Gross A, Terraza A, Ouahrani-Bettache S, Liautard JP, Dornand J. 2000. In vitro *Brucella suis* infection prevents the programmed cell death of human monocytic cells. Infect Immun 68:342–351. doi:10.1128/iai.68.1.342-351.2000. 10603407 PMC97140

[18] Choi KS, Park JT, Dumler JS. 2005. *Anaplasma phagocytophilum* delay of neutrophil apoptosis through the p38 mitogen-activated protein kinase signal pathway. Infect Immun 73:8209–8218. doi:10.1128/IAI.73.12.8209-8218.2005. 16299317 PMC1307085

[19] Clark CS, Maurelli AT. 2007. *Shigella flexneri* inhibits staurosporine-induced apoptosis in epithelial cells. Infect Immun 75:2531–2539. doi:10.1128/IAI.01866-06. 17339354 PMC1865761

[20] Falkow S, Isberg RR, Portnoy DA. 1992. The interaction of bacteria with mammalian cells. Annu Rev Cell Biol 8:333–363. doi:10.1146/annurev.cb.08.110192.002001. 1476803

[21] Ernst RK, Guina T, Miller SI. 1999. How intracellular bacteria survive: surface modifications that promote resistance to host innate immune responses. J Infect Dis 179:S326–S330. doi:10.1086/513850. 10081503

[22] Niu H, Kozjak-Pavlovic V, Rudel T, Rikihisa Y. 2010. *Anaplasma phagocytophilum* Ats-1 is imported into host cell mitochondria and interferes with apoptosis induction. PLoS Pathog 6:e1000774. doi:10.1371/journal.ppat.1000774. 20174550 PMC2824752

[23] Ge Y, Rikihisa Y. 2006. *Anaplasma phagocytophilum* delays spontaneous human neutrophil apoptosis by modulation of multiple apoptotic pathways. Cell Microbiol 8:1406–1416. doi:10.1111/j.1462-5822.2006.00720.x. 16922860

[24] Clifton DR, Goss RA, Sahni SK, Van Antwerp D, Baggs RB, Marder VJ, Silverman DJ, Sporn LA. 1998. NF-κB-dependent inhibition of apoptosis is essential for host cell survival during *Rickettsia rickettsii* infection. Proc Natl Acad Sci U S A 95:4646–4651. doi:10.1073/pnas.95.8.4646. 9539792 PMC22544

[25] Joshi SG, Francis CW, Silverman DJ, Sahni SK. 2004. NF-κB activation suppresses host cell apoptosis during *Rickettsia rickettsii* infection via regulatory effects on intracellular localization or levels of apoptogenic and anti-apoptotic proteins. FEMS Microbiol Lett 234:333–341. doi:10.1016/j.femsle.2004.03.046. 15135541

[26] Bechelli JR, Rydkina E, Colonne PM, Sahni SK. 2009. *Rickettsia rickettsii* infection protects human microvascular endothelial cells against staurosporine-induced apoptosis by a cIAP2-independent mechanism. J Infect Dis 199:1389–1398. doi:10.1086/597805. 19358671

[27] L’amoreaux WJ, Junaid L, Trevidi S. 2003. Morphological evidence that salivary gland degeneration in the American dog tick, *Dermacentor variabilis* (Say), involves programmed cell death. Tissue Cell 35:95–99. doi:10.1016/S0040-8166(02)00109-X. 12747931

[28] Freitas DR, Rosa RM, Moura DJ, Seitz AL, Colodel EM, Driemeier D, Vaz ID, Jr, Masuda A. 2007. Cell death during preoviposition period in *Boophilus microplus* tick. Vet Parasitol 144:321–327. doi:10.1016/j.vetpar.2006.10.017. 17157985

[29] Ayllón N, Villar M, Busby AT, Kocan KM, Blouin EF, Bonzón-Kulichenko E, Galindo RC, Mangold AJ, Alberdi P, Pérez de la Lastra JM, Vázquez J, de la Fuente J. 2013. *Anaplasma phagocytophilum* inhibits apoptosis and promotes cytoskeleton rearrangement for infection of tick cells. Infect Immun 81:2415–2425. doi:10.1128/IAI.00194-13. 23630955 PMC3697600

[30] Ayllón N, Villar M, Galindo RC, Kocan KM, Šíma R, Lopez JA, Vazquez J, Alberdi P, Cabezas-Cruz A, Kopáček P, De La Fuente J. 2015. Systems biology of tissue-specific response to *Anaplasma phagocytophilum* reveals differentiated apoptosis in the tick vector *Ixodes scapularis*. PLoS Genet 11:e1005120. doi:10.1371/journal.pgen.1005120. 25815810 PMC4376793

[31] Wang Y, Hu S, Tuerdi M, Yu X, Zhang H, Zhou Y, Cao J, da Silva Vaz I, Zhou J. 2020. Initiator and executioner caspases in salivary gland apoptosis of *Rhipicephalus haemaphysaloides*. Parasit Vectors 13:1–15. doi:10.1186/s13071-020-04164-5. 32503655 PMC7275347

[32] Paddock CD, Sumner JW, Comer JA, Zaki SR, Goldsmith CS, Goddard J, McLellan SL, Tamminga CL, Ohl CA. 2004. *Rickettsia parkeri*: a newly recognized cause of spotted fever rickettsiosis in the United States. Clin Infect Dis 38:805–811. doi:10.1086/381894. 14999622

[33] Serio AW, Jeng RL, Haglund CM, Reed SC, Welch MD. 2010. Defining a core set of actin cytoskeletal proteins critical for actin-based motility of Rickettsia. Cell Host Microbe 7:388–398. doi:10.1016/j.chom.2010.04.008. 20478540 PMC2935136

[34] Paddock CD, Finley RW, Wright CS, Robinson HN, Schrodt BJ, Lane CC, Ekenna O, Blass MA, Tamminga CL, Ohl CA, McLellan SLF, Goddard J, Holman RC, Openshaw JJ, Sumner JW, Zaki SR, Eremeeva ME. 2008. *Rickettsia parkeri* rickettsiosis and its clinical distinction from Rocky Mountain spotted fever. Clin Infect Dis 47:1188–1196. doi:10.1086/592254. 18808353

[35] Zhang Y, Chen X, Gueydan C, Han J. 2018. Plasma membrane changes during programmed cell deaths. Cell Res 28:9–21. doi:10.1038/cr.2017.133. 29076500 PMC5752838

[36] Wlodkowic D, Telford W, Skommer J, Darzynkiewicz Z. 2011. Apoptosis and beyond: cytometry in studies of programmed cell death. Methods Cell Biol 103:55–98. doi:10.1016/B978-0-12-385493-3.00004-8. 21722800 PMC3263828

[37] Wright CL, Sonenshine DE, Gaff HD, Hynes WL. 2015. *Rickettsia parkeri* transmission to *Amblyomma americanum* by cofeeding with *Amblyomma maculatum* (Acari: Ixodidae) and potential for spillover. J Med Entomol 52:1090–1095. doi:10.1093/jme/tjv086. 26336226 PMC12119055

[38] Shi Y. 2004. Caspase activation, inhibition, and reactivation: a mechanistic view. Protein Sci 13:1979–1987. doi:10.1110/ps.04789804. 15273300 PMC2279816

[39] Garrido C, Galluzzi L, Brunet M, Puig PE, Didelot C, Kroemer G. 2006. Mechanisms of cytochrome c release from mitochondria. Cell Death Differ 13:1423–1433. doi:10.1038/sj.cdd.4401950. 16676004

[40] Adams JM, Cory S. 2001. Life-or-death decisions by the Bcl-2 protein family. Trends Biochem Sci 26:61–66. doi:10.1016/s0968-0004(00)01740-0. 11165519

[41] Sharma R, Ahmad G, Esteves SC, Agarwal A. 2016. Terminal deoxynucleotidyl transferase dUTP nick end labeling (TUNEL) assay using bench top flow cytometer for evaluation of sperm DNA fragmentation in fertility laboratories: protocol, reference values, and quality control. J Assist Reprod Genet 33:291–300. doi:10.1007/s10815-015-0635-7. 26780327 PMC4758999

[42] Thakur A, Mikkelsen H, Jungersen G. 2019. Intracellular pathogens: host immunity and microbial persistence strategies. J Immunol Res 2019:1356540. doi:10.1155/2019/1356540. 31111075 PMC6487120

[43] Fan T, Lu H, Hu H, Shi L, McClarty GA, Nance DM, Greenberg AH, Zhong G. 1998. Inhibition of apoptosis in chlamydia-infected cells: blockade of mitochondrial cytochrome c release and caspase activation. J Exp Med 187:487–496. doi:10.1084/jem.187.4.487. 9463399 PMC2212145

[44] Ojcius DM, Souque P, Perfettini JL, Dautry-Varsat A. 1998. Apoptosis of epithelial cells and macrophages due to infection with the obligate intracellular pathogen *Chlamydia psittaci*. J Immunol Res 161:4220–4226. 9780196

[45] Goyal G, Fell B, Sarin A, Youle RJ, Sriram V. 2007. Role of mitochondrial remodeling in programmed cell death in *Drosophila melanogaster*. Dev Cell 12:807–816. doi:10.1016/j.devcel.2007.02.002. 17488630 PMC1885957

[46] Jamil S, Lam I, Majd M, Tsai S-H, Duronio V. 2015. Etoposide induces cell death via mitochondrial-dependent actions of p53. Cancer Cell Int 15:79. doi:10.1186/s12935-015-0231-z. 26251638 PMC4527242

[47] Wang XR, Wang C, Ban FX, Ghanim M, Pan LL, Qian LX, Liu YQ, Wang XW, Liu SS. 2020. Apoptosis in a whitefly vector activated by a begomovirus enhances viral transmission. mSystems 5:e00433. doi:10.1128/mSystems.00433-20. 32963100 PMC7511215

[48] Taylor RC, Cullen SP, Martin SJ. 2008. Apoptosis: controlled demolition at the cellular level. Nat Rev Mol Cell Biol 9:231–241. doi:10.1038/nrm2312. 18073771

[49] Sivandzade F, Bhalerao A, Cucullo L. 2019. Analysis of the mitochondrial membrane potential using the cationic JC-1 dye as a sensitive fluorescent probe. Bio Protoc 9:e3128. doi:10.21769/BioProtoc.3128. PMC6343665 30687773

[50] Dunn KW, Kamocka MM, McDonald JH. 2011. A practical guide to evaluating colocalization in biological microscopy. Am J Physiol Cell Physiol 300:C723–C742. doi:10.1152/ajpcell.00462.2010. 21209361 PMC3074624

[51] Aaron JS, Taylor AB, Chew TL. 2019. The Pearson's correlation coefficient is not a universally superior colocalization metric. Response to ‘Quantifying colocalization: the MOC is a hybrid coefficient–an uninformative mix of co-occurrence and correlation’. J Cell Sci 132:jcs227074. doi:10.1242/jcs.227074. 30626690

[52] Maurin M, Raoult D. 2001. Use of aminoglycosides in treatment of infections due to intracellular bacteria. Antimicrob Agents Chemother 45:2977–2986. doi:10.1128/AAC.45.11.2977-2986.2001. 11600345 PMC90771

[53] Burkhardt NY, Baldridge GD, Williamson PC, Billingsley PM, Heu CC, Felsheim RF, Kurtti TJ, Munderloh UG. 2011. Development of shuttle vectors for transformation of diverse *Rickettsia* species. PLoS One 6:e29511. doi:10.1371/journal.pone.0029511. 22216299 PMC3244465

[54] Shin S, Roy CR. 2008. Host cell processes that influence the intracellular survival of Legionella pneumophila. Cell Microbiol 10:1209–1220. doi:10.1111/j.1462-5822.2008.01145.x. 18363881

[55] Alberdi P, Mansfield KL, Manzano-Román R, Cook C, Ayllón N, Villar M, Johnson N, Fooks AR, de la Fuente J. 2016. Tissue-specific signatures in the transcriptional response to *Anaplasma phagocytophilum* infection of *Ixodes scapularis* and *Ixodes ricinus* tick cell lines. Front Cell Infect Microbiol 6:20. doi:10.3389/fcimb.2016.00020. 26904518 PMC4748044

[56] Aliprantis AO, Yang RB, Mark MR, Suggett S, Devaux B, Radolf JD, Klimpel GR, Godowski P, Zychlinsky A. 1999. Cell activation and apoptosis by bacterial lipoproteins through Toll-like receptor-2. Science 285:736–739. doi:10.1126/science.285.5428.736. 10426996

[57] Rojas M, Barrera LF, Puzo G, Garcia LF. 1997. Differential induction of apoptosis by virulent Mycobacterium tuberculosis in resistant and susceptible murine macrophages: role of nitric oxide and mycobacterial products. J Immunol Res 159:1352–1361. 9233632

[58] Oliver JD, Chávez AS, Felsheim RF, Kurtti TJ, Munderloh UG. 2015. An Ixodes scapularis cell line with a predominantly neuron-like phenotype. Exp Appl Acarol 66:427–442. doi:10.1007/s10493-015-9908-1. 25894426 PMC4449809

[59] Liu KY, Yang H, Peng JX, Hong HZ. 2012. Cytochrome c and insect cell apoptosis. Insect Sci 19:30–40. doi:10.1111/j.1744-7917.2011.01431.x.

[60] Dorstyn L, Mills K, Lazebnik Y, Kumar S. 2004. The two cytochrome c species, DC3 and DC4, are not required for caspase activation and apoptosis in *Drosophila* cells. J Cell Biol 167:405–410. doi:10.1083/jcb.200408054. 15533997 PMC2172470

[61] Kumarswamy R, Seth RK, Dwarakanath BS, Chandna S. 2009. Mitochondrial regulation of insect cell apoptosis: evidence for permeability transition pore‐independent cytochrome‐c release in the *Lepidopteran* Sf9 cells. Int J Biochem Cell Biol 41:1430–1440. doi:10.1016/j.biocel.2008.12.009. 19146980

[62] Facey CO, Lockshin RA. 2010. The execution phase of autophagy associated PCD during insect metamorphosis. Apoptosis 15:639–652. doi:10.1007/s10495-010-0499-3. 20405221

[63] Hakim RS, Baldwin K, Smagghe G. 2010. Regulation of midgut growth, development and metamorphosis. Annu Rev Entomol 55:593–608. doi:10.1146/annurev-ento-112408-085450. 19775239

[64] Lehman SS, Noriea NF, Aistleitner K, Clark TR, Dooley CA, Nair V, Kaur SJ, Rahman MS, Gillespie JJ, Azad AF, Hackstadt T. 2018. The rickettsial ankyrin repeat protein 2 is a type IV secreted effector that associates with the endoplasmic reticulum. mBio 9:e00975-18. doi:10.1128/mBio.00975-18. 29946049 PMC6020290

[65] Ulett GC, Adderson EE. 2006. Regulation of apoptosis by gram-positive bacteria: mechanistic diversity and consequences for immunity. Curr Immunol Rev 2:119–141. doi:10.2174/157339506776843033. 19081777 PMC2600511

[66] Rennoll-Bankert KE, Rahman MS, Gillespie JJ, Guillotte ML, Kaur SJ, Lehman SS, Beier-Sexton M, Azad AF. 2015. Which way in? The RalF Arf-GEF orchestrates Rickettsia host cell invasion. PLoS Pathog 11:e1005115. doi:10.1371/journal.ppat.1005115. 26291822 PMC4546372

[67] Curto P, Riley SP, Simões I, Martinez JJ. 2019. Macrophages infected by a pathogen and a non-pathogen spotted fever group Rickettsia reveal differential reprogramming signatures early in infection. Front Cell Infect Microbiol 9:97. doi:10.3389/fcimb.2019.00097. 31024862 PMC6467950

[68] Burgdorfer W, Brinton LP. 1975. Mechanisms of transovarial infection of spotted fever rickettsiae in ticks. Ann N Y Acad Sci 266:61–72. doi:10.1111/j.1749-6632.1975.tb35088.x. 829476

[69] Azad AF, Beard CB. 1998. Rickettsial pathogens and their arthropod vectors. Emerg Infect Dis 4:179–186. doi:10.3201/eid0402.980205. 9621188 PMC2640117

[70] Niebylski ML, Peacock MG, Schwan TG. 1999. Lethal effect of *Rickettsia rickettsii* on its tick vector (*Dermacentor andersoni*). Appl Environ Microbiol 65:773–778. doi:10.1128/AEM.65.2.773-778.1999. 9925615 PMC91094

[71] Schumacher L, Snellgrove A, Levin ML. 2016. Effect of *Rickettsia rickettsii* (Rickettsiales: Rickettsiaceae) infection on the biological parameters and survival of its tick vector—*Dermacentor variabilis* (Acari: Ixodidae). J Med Entomol 53:172–176. doi:10.1093/jme/tjv166. 26494822 PMC5659120

[72] Yang X, Koči J, Smith AA, Zhuang X, Sharma K, Dutta S, Rana VS, Kitsou C, Yas OB, Mongodin EF, Pal U. 2021. A novel tick protein supports integrity of gut peritrophic matrix impacting existence of gut microbiome and Lyme disease pathogens. Cell Microbiol 23:e13275. doi:10.1111/cmi.13275. 33006213 PMC9038076

[73] Wang XR, Kurtti TJ, Oliver JD, Munderloh UG. 2020. The identification of tick autophagy-related genes in *Ixodes scapularis* responding to amino acid starvation. Ticks Tick Borne Dis 11:101402. doi:10.1016/j.ttbdis.2020.101402. 32035896 PMC7127957

[74] Kurtti TJ, Simser JA, Baldridge GD, Palmer AT, Munderloh UG. 2005. Factors influencing in vitro infectivity and growth of *Rickettsia peacockii* (Rickettsiales: Rickettsiaceae), an endosymbiont of the Rocky Mountain wood tick, *Dermacentor andersoni* (Acari, Ixodidae). J Invertebr Pathol 90:177–186. doi:10.1016/j.jip.2005.09.001. 16288906 PMC1625098

[75] Stenos J, Graves SR, Unsworth NB. 2005. A highly sensitive and specific real-time PCR assay for the detection of spotted fever and typhus group Rickettsiae. Am J Trop Med Hyg 73:1083–1085. 16354816

