# Supplementary figures and images for: Mitochondrion-Dependent Apoptosis Is Essential for Rickettsia parkeri Infection and Replication in Vector Cells
Source: mSystems. 2021 Mar 16;6(2):e01209-20. doi: 10.1128/mSystems.01209-20 (PMC8546998; doi:10.1128/mSystems.01209-20)

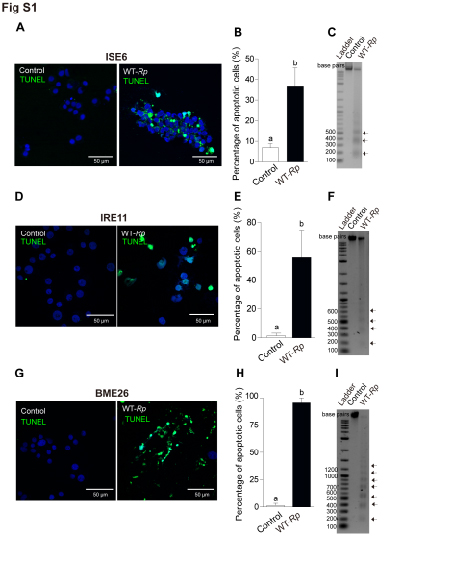

Supplement: FIG S1 [file msystems.01209-20-sf001.jpg]

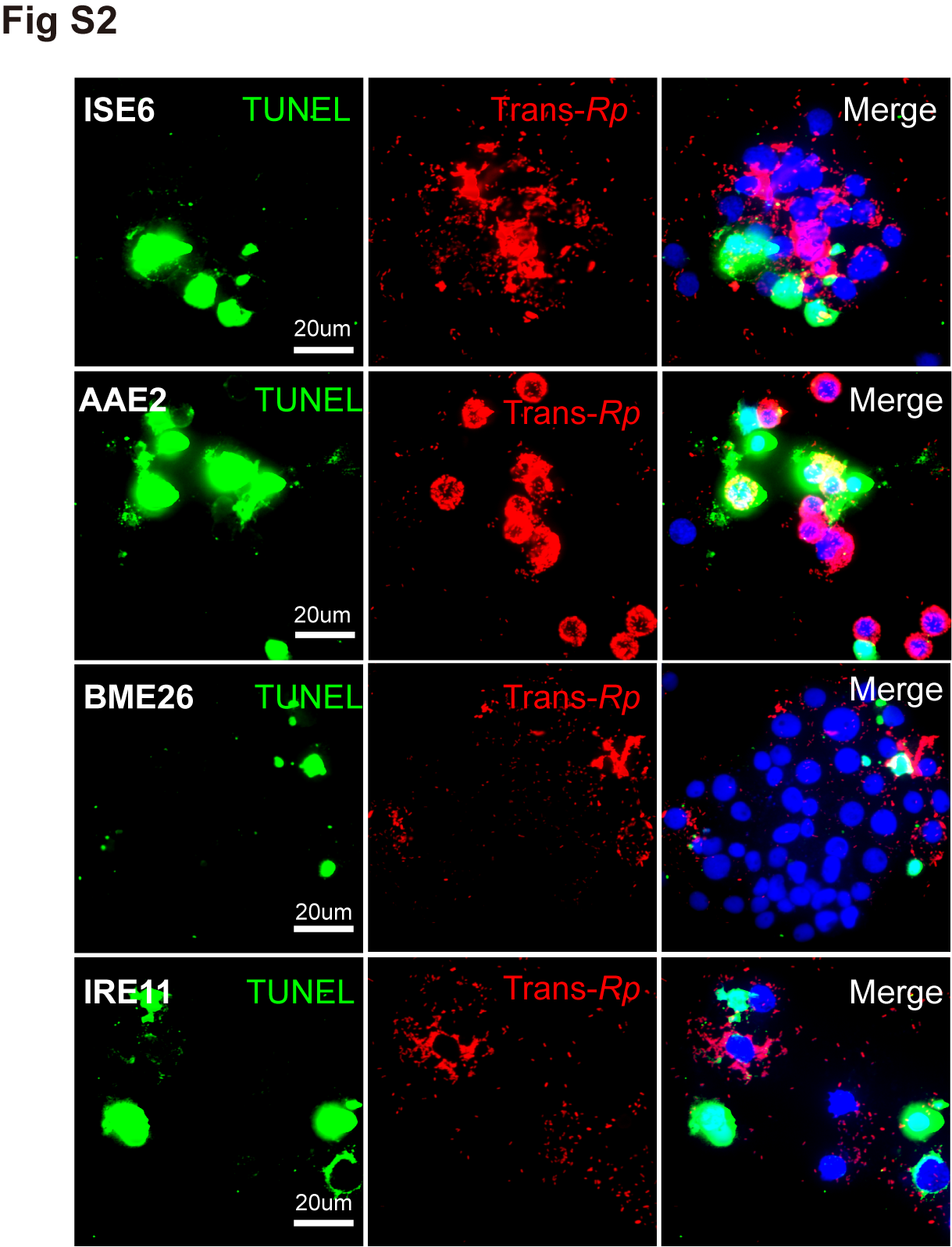

Supplement: FIG S2 [file msystems.01209-20-sf002.tif]

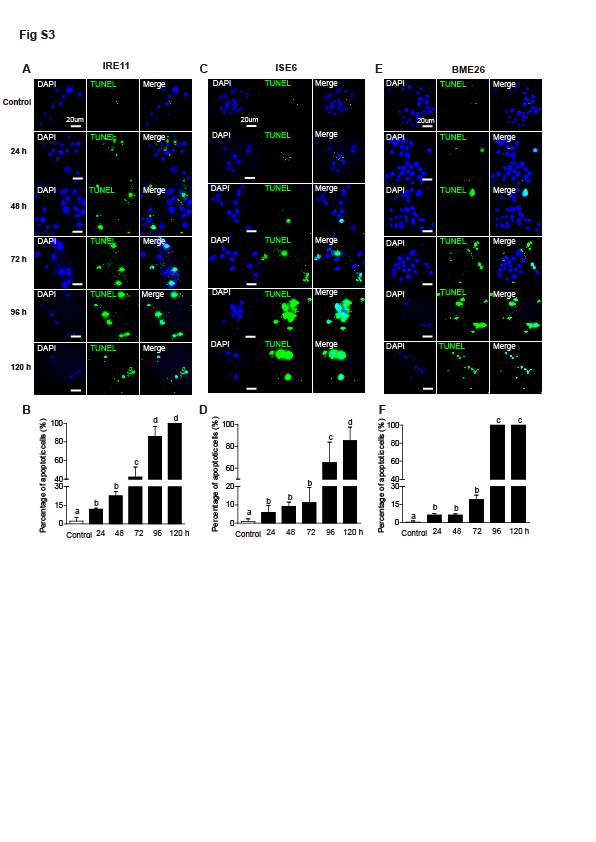

Supplement: FIG S3 [file msystems.01209-20-sf003.jpg]

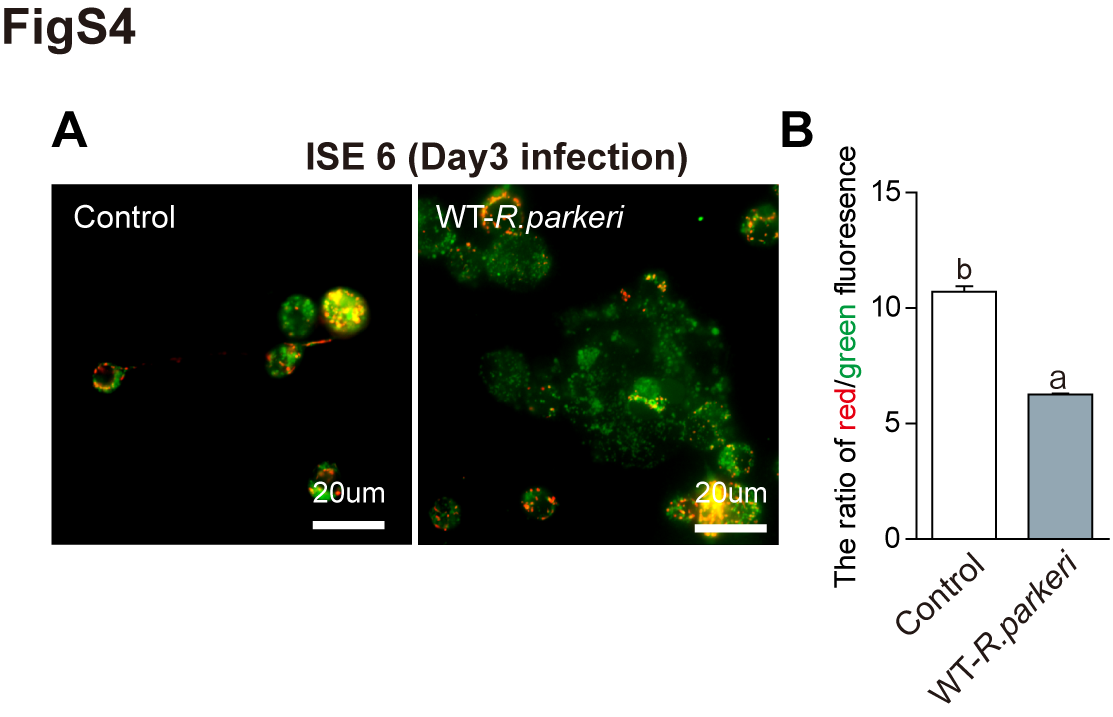

Supplement: FIG S4 [file msystems.01209-20-sf004.tif]

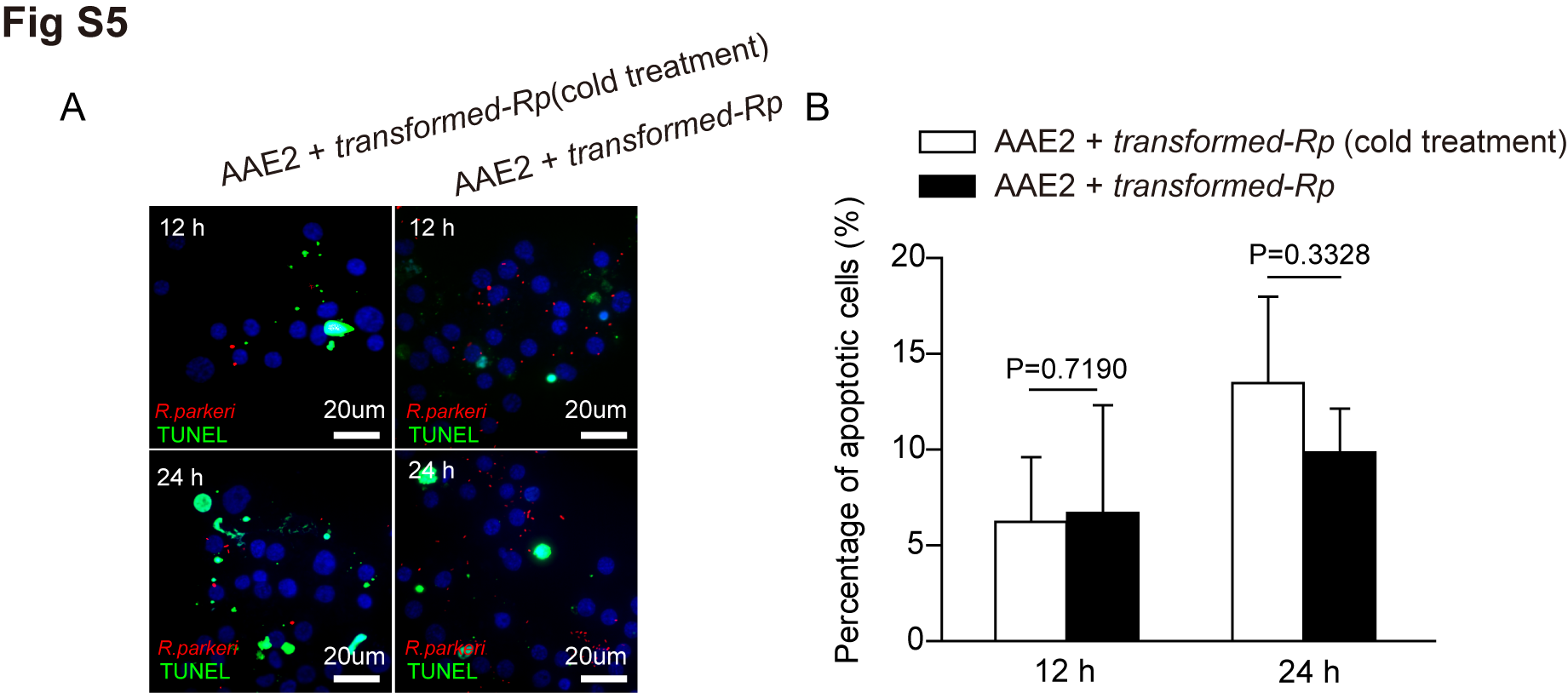

Supplement: FIG S5 [file msystems.01209-20-sf005.tif]

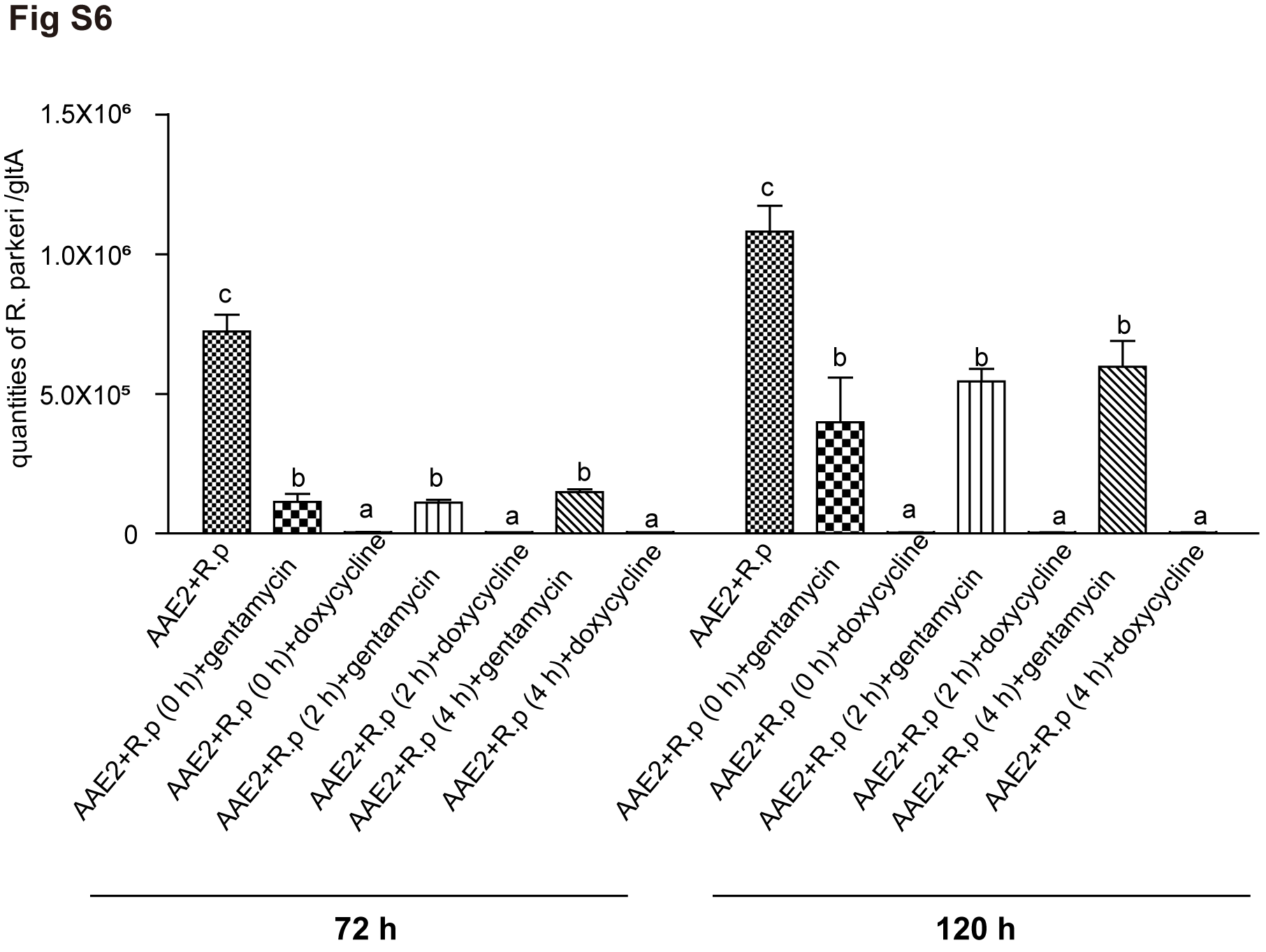

Supplement: FIG S6 [file msystems.01209-20-sf006.tif]

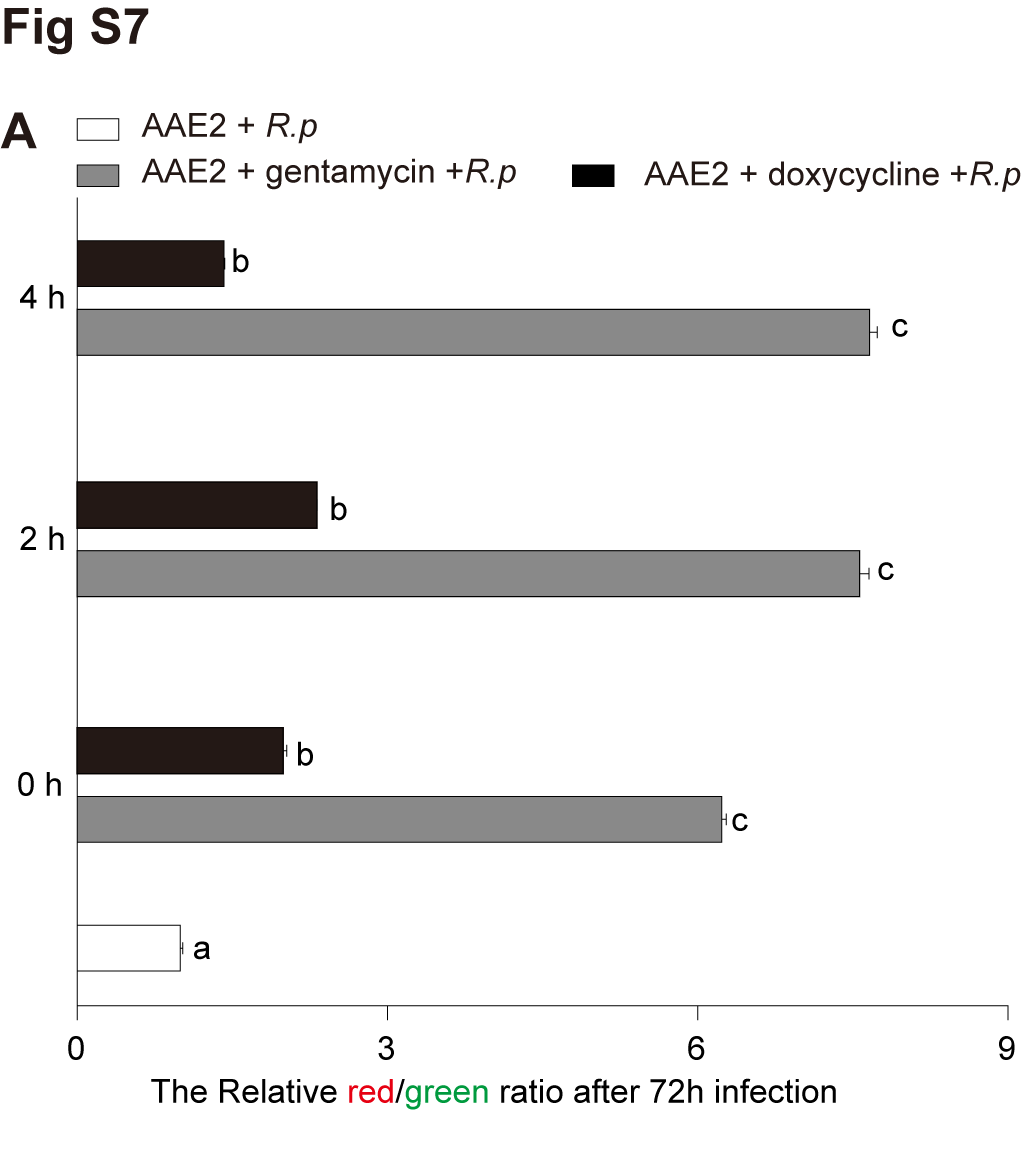

Supplement: FIG S7 [file msystems.01209-20-sf007.tif]

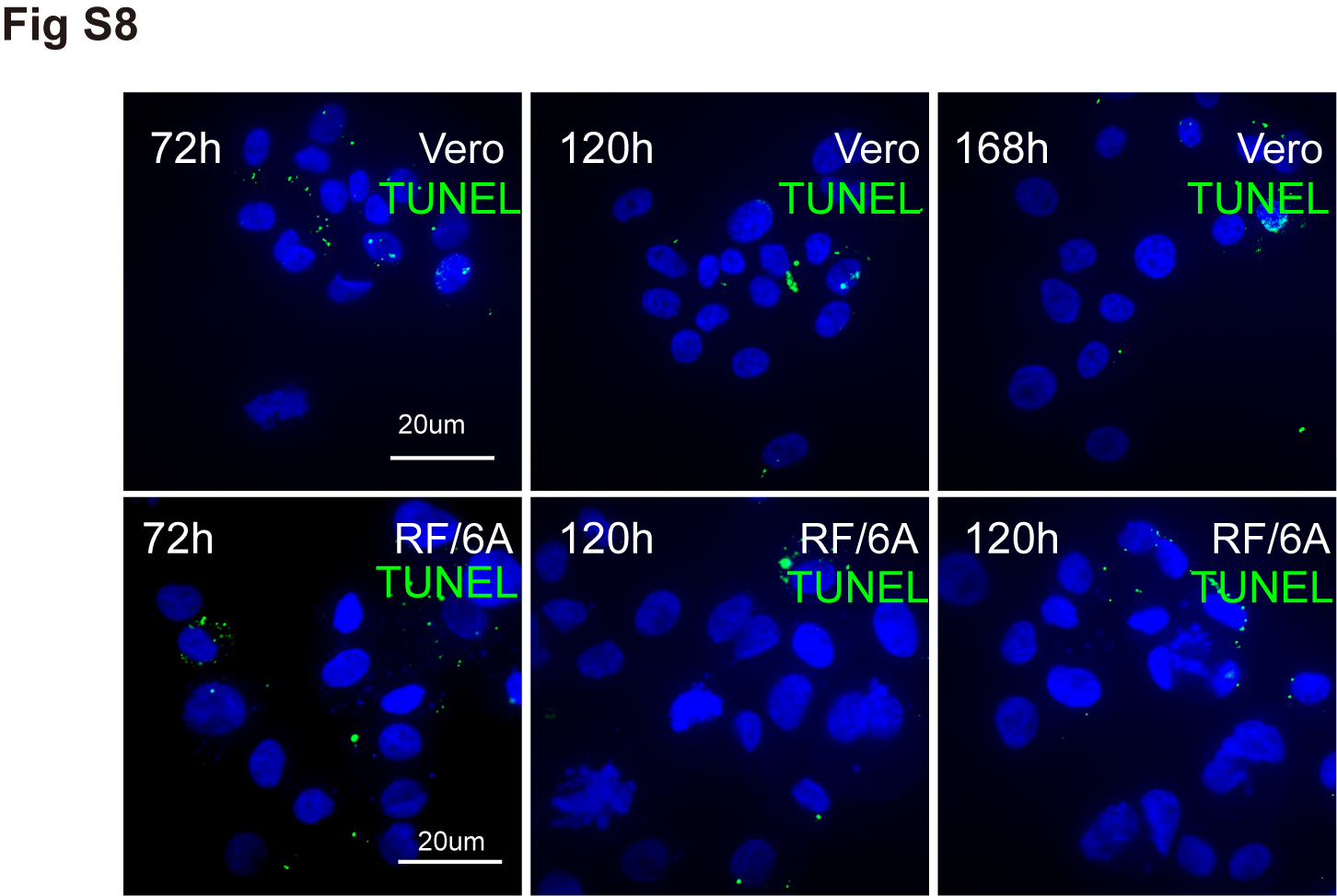

Supplement: FIG S8 [file msystems.01209-20-sf008.tif]
