# Supplementary material for: Mitochondrion-Dependent Apoptosis Is Essential for Rickettsia parkeri Infection and Replication in Vector Cells
Source: mSystems. 2021 Mar 16;6(2):e01209-20. doi: 10.1128/mSystems.01209-20 (PMC8546998; doi:10.1128/mSystems.01209-20)
Supplement: TABLE S1 [file msystems.01209-20-st001.docx]

**Supplementary Table S1. SFG *Rickettsia* species, strains used in this study, related to Methods.**

| SFG species and strains | Vector tick |
| --- | --- |
| *Rickettsia parkeri* Tate’s Hell  *Rickettsia monacensis* IrR Munich | *Amblyomma maculatum*  *Ixodes ricinus* |
| *Rickettsia helvetica* C9P9  *Rickettsia. amblyommatis* AaR/SC | *Ixodes ricinus*  *Amblyomma americanum.* |
