# Supplementary material for: Mitochondrion-Dependent Apoptosis Is Essential for Rickettsia parkeri Infection and Replication in Vector Cells
Source: mSystems. 2021 Mar 16;6(2):e01209-20. doi: 10.1128/mSystems.01209-20 (PMC8546998; doi:10.1128/mSystems.01209-20)
Supplement: TABLE S2 [file msystems.01209-20-st002.docx]

| Gene name | Forward primer (5’–3’) | Reverse primer (5’–3’) |
| --- | --- | --- |
| *caspase1*  *caspase3*  *cytochrome c* | AAACAAAGGACAACGCCGAC  AGTGAAAGGAGGGGTACCGA  AGAGCAGCCATGGTCGAAAT | GTCCTTGTAATCCGTGCCGT  CTCAGGATGCAGCAGACGAA  TCTGTGTAGCTGAAGCCTGC |
| *bcl-2* | TTTGCTCGCATACCGAGGTC | TACGACGAGATGCACGCCACA |
| *iap*  *gapdh*  *gltA (rickettsia)* | AACTCCCACTTGAAGATGGC  ATTGGAGACACCCACAGCAG TCGCAAATGTTCACGGTACTTT | CATGGTCGGAGACACCTGG  GACACGCTTCACTGGTCCTT TCGTGCATTTCTTTCCATTGTG |

**Supplementary Table S2. qRT-PCR primers, related to Methods.**
